# Meta-analysis of larvae of the black soldier fly (*Hermetia illucens*) microbiota based on 16S rRNA gene amplicon sequencing

**DOI:** 10.1093/femsec/fiac094

**Published:** 2022-08-17

**Authors:** Freek IJdema, Jeroen De Smet, Sam Crauwels, Bart Lievens, Leen Van Campenhout

**Affiliations:** CLMT Research Group for Insect Production and Processing, Department of Microbial and Molecular Systems (M^2^S), KU Leuven, B-3001, Campus Geel, Geel, B-2440, Belgium; KU Leuven, Leuven Food Science and Nutrition Research Centre (LFoRCe), Leuven, B-3001, Belgium; CLMT Research Group for Insect Production and Processing, Department of Microbial and Molecular Systems (M^2^S), KU Leuven, B-3001, Campus Geel, Geel, B-2440, Belgium; KU Leuven, Leuven Food Science and Nutrition Research Centre (LFoRCe), Leuven, B-3001, Belgium; CMPG Laboratory for Process Microbial Ecology and Bioinspirational Management (PME&BIM), Department of Microbial and Molecular Systems (M²S), KU Leuven, Leuven, B-3001, Belgium; CMPG Laboratory for Process Microbial Ecology and Bioinspirational Management (PME&BIM), Department of Microbial and Molecular Systems (M²S), KU Leuven, Leuven, B-3001, Belgium; Leuven Plant Institute (LPI), KU Leuven, Leuven, B-3001, Belgium; CLMT Research Group for Insect Production and Processing, Department of Microbial and Molecular Systems (M^2^S), KU Leuven, B-3001, Campus Geel, Geel, B-2440, Belgium; KU Leuven, Leuven Food Science and Nutrition Research Centre (LFoRCe), Leuven, B-3001, Belgium

**Keywords:** bacteria, bio-informatics, composition, core, edible insect, gut

## Abstract

Black soldier fly larvae (BSFL) belong to the most widely reared insects as an alternative protein source at industrial scale. Bacteria in the larval gut can provide benefits for the animal, though some bacteria can also be pathogenic for the insect. Accurate characterization of the BSFL microbiota is important for the production of BSFL in terms of yield and microbiological safety. In this study, 16S ribosomal RNA gene sequence data sets from 11 studies were re-analysed to gain better insights in the BSFL gut microbiota, potential factors that influence their composition, and differences between the gut and the whole larvae microbiota. A core gut microbiota was found consisting of members of *Enterococcus*, *Klebsiella, Morganella*, *Providencia*, and *Scrofimicrobium*. Further, the factors ‘Study’, ‘Age’ and ‘Feed’ (*i.e*. rearing substrate of the larvae) significantly affected the microbiota gut composition. When compared to whole larvae, a significantly lower diversity was found for gut samples, suggesting that the larvae harboured additional microbes on their cuticle or in the insect body. Universal choices in insect sample type, primer selection and bio-informatics analysis pipeline can strengthen future meta-analyses and improve our understanding of the BSFL gut microbiota towards the optimization of insect rearing conditions and substrates.

## Teaser

Through a meta-analysis of 16S rRNA gene sequencing data, this study identifies the core gut microbiota in BSFL and explores how experimental parameters and bio-informatic pipeline construction impact its composition.

## Introduction

Insects are one of the most promising alternative sources to sustainably produce proteins. They not only contain high quality protein and essential nutrients, they also have a high potential for efficient upgrading of waste streams. In this regard, black soldier fly larvae (BSFL) (*Hermetia illucens* L., Diptera: Stratiomyidae) are one of the most promising insects to be used as an alternative protein source in animal feeds. The species, originally traced to the Americas, occurs in most tropical and temperate regions of the world (Sheppard et al. 1994). Although their nutritional value varies, crude proteins of BSFL can account for 32–45% of the total dry weight of the larvae (Diener et al. 2009, Caligiani et al. 2018), while fat constitutes about 15–49% of their total dry weight (Makkar et al. 2014). In addition, their amino acid profile is similar to that of fishmeal (Belghit et al. 2019). Though naturally occurring in chicken, pig and cow manure, BSFL have been successfully reared on diverse organic waste streams and are being cultivated at industrial scale (Miranda et al. 2020, Tomberlin and Van Huis 2020, Yang & Tomberlin, 2020). Altogether, this makes BSFL a highly suitable alternative for traditional animal protein.

Microbes in the gut can substantially support nutrient uptake processes and affect the host immune system (O'Hara et al. 2020). The gut microbiota has therefore been an important subject of study in animal health and production (Holman and Gzyl 2019, O'Hara et al. 2020), and likewise, it is useful to investigate the microbiota in insect production. Several studies have investigated the microbiota in and on BSFL, aiming to get a better understanding of the BSFL microbial community composition and function to aid their industrial production, as well as to assess potential microbial hazards associated with their consumption (Wynants et al. 2019, Khamis et al. 2020). The dissected insect gut is often used for microbiota analysis, although some studies have also focused on whole larvae. The choice for a specific sample type can depend on the focus of the study. However, there might be a difference between the microbiota composition of both sample types. Aside from the insect gut, microorganisms can be present in the insect haemolymph (Aronson et al. 1986) or on the surface of the insect (Ulanova et al. 2020). External bacteria could enter the larvae through oral ingestion but also through wounds or pores in the insect cuticle (Joosten et al. 2020). It is therefore important not only to consider the gut microbiota, but also to take into account microorganisms colonizing other parts in the insect.

Whereas bacterial communities have been traditionally studied through culture-based methods, focus has shifted towards next-generation sequencing approaches that rely on sequencing of a taxonomic marker such as the 16S ribosomal RNA (rRNA) gene. In general, the method uses PCR to multiply the marker of interest, after which amplicons from different samples are pooled and sequenced. Compared to culture-dependent methods, sequencing-based approaches have several advantages (Hiergeist et al. 2015). It is well known that not all microorganisms can be cultivated in standard laboratory conditions. Furthermore, sometimes important microbial groups are missed due to culture-dependent biases, such as the appearance of microorganisms in a viable but non-culturable state and/or the possibility that isolation media favor the cultivation of specific microorganisms, that outcompete others. Additionally, microorganisms may remain undetected when they occur at cell densities below the limit of detection of the plate methods. These drawbacks can be circumvented by next-generation amplicon sequencing technologies, enabling an in-depth characterization of microbial communities without culturing (Oulas et al. 2015).

In general, 16 phyla of bacteria have been identified in the BSFL gut, with Firmicutes, Bacteroidetes and Proteobacteria being the most dominant phyla (Huang et al. 2020, Zhan et al. 2020, Zhang et al. 2020). Ao et al. (2020) found that the amount of total nitrogen and fat was positively correlated with the relative abundance of *Providencia* in the gut of BSFL fed on chicken and swine manure. This suggests that *Providencia* might play an important role in protein and lipid conversion in the gut (Ao et al. 2020). *Providencia* was also found at high relative abundance in other studies focussing on BSFL microbiota (Zheng et al. 2013a, Wynants et al. 2019, Raimondi et al. 2020, Gorrens et al. 2021). Additionally, Bruno et al. (2019) suggested that members of the genera *Sphingobacterium* and *Dysgonomonas* may play a role in the degradation of polysaccharides in the BSFL gut. In contrast, there are also bacteria in the BSFL gut which may have a deleterious effect on their host. For example, Wu et al. (2020) found members of bacterial families in the BSFL gut that may be detrimental, including the families *Brucellaceae* and *Enterobacteriaceae*, and the genus *Campylobacter*. Zheng et al. (2013a) found potential pathogenic *Enterobacteriaceae* members in all life stages of BSFL raised on chicken feed. Further, it has been found that environmental factors, such as rearing temperature and diet, have a strong impact on the BSFL microbial community composition and diversity. Raimondi et al. (2020) found a negative correlation between temperature and the relative abundance of *Providencia*, but a positive correlation with several other bacteria (*e.g. Alcaligenes, Pseudogracilibacillus, Proteus*). For many insect species it has been shown that diet has a large impact on the gut microbiota (Engel and Moran 2013). This has also been demonstrated for BSFL (Bruno et al. 2019, Ao et al. 2020). Furthermore, studies have suggested that geographic origin and the in-house microbiota of rearing facilities may influence the microbial community composition of edible insects (Khamis et al. 2020, Wynants et al. 2019).

Although our knowledge on BSFL microbiota and potential factors determining their microbial community composition has clearly increased in recent years, it remains challenging to identify general trends and draw general conclusions over the different studies performed. Studies have been performed using different PCR primers spanning different regions of the 16S rRNA gene, which may affect the outcome of microbiota studies (Albertsen et al. 2015, Tremblay et al. 2015, Fouhy et al. 2016, Rintala et al. 2017, Zhang et al. 2018). Additionally, different bioinformatics data analysis pipelines have been used to analyse the data (e.g. MOTHUR, QIIME, USEARCH) with various workflows. Moreover, there is a growing tendency to replace the use of operational taxonomic units (OTUs) based on a sequence similarity cut-off by the analysis of exact sequence variants, i.e. so-called ‘amplicon sequence variants’ (ASVs) (also termed ‘zero-radius OTUs’ (zOTUs)) (Callahan et al. 2017; Edgar, 2016), increasing taxonomic resolution. Altogether, this makes it very difficult to compare different studies. Therefore, to increase our understanding of the BSFL microbiota and the underlying mechanisms shaping the microbial communities, a profound meta-analysis of the different studies performed is needed, in which all data is analysed in the same manner.

In this study, the BSFL gut microbiota is characterized in detail by re-processing raw 16S rRNA gene sequencing data from different publicly available studies through the same bioinformatic pipeline. A first objective was to investigate whether a specific set of gut bacteria can be identified despite varying experimental conditions (the ‘core’ microbiota), and if so, which genera or species belong to the core gut microbiota. Secondly, we aimed to study which factors, such as type of feed and age of the larvae at harvest, shape the bacterial community composition in BSFL gut samples. Finally, a comparison was made between the microbiota composition found in the gut of BSFL and in larvae as a whole to investigate differences between these two sample types.

## Materials and methods

### Data acquisition and quality filters

A total of 11 studies were included in this meta-analysis (Table 1). The studies were retrieved through a literature search (performed in 2021) using a combination of the key words ‘*microbiome*’, ‘*black soldier fly*’, ‘*microbiota*’, and ‘*Hermetia illucens*’ in Scopus, PubMed and Google Scholar. Additionally, potential datasets were identified through a search in the Sequence Read Archive (SRA) database from NCBI (available from: http://www.ncbi.nlm.nih.gov/pubmed) by entering the key words ‘*black soldier fly*’ or ’*Hermetia illucens’* (accessed in 2021), resulting in 40 and 41 BioProject hits, respectively. To be included in the meta-analysis, all studies were required (i) to have analysed the BSFL microbiota, (ii) to have analysed samples that reflect a ‘natural’, inartificial state of the microbiota composition, which excludes studies where bacterial strains were injected or introduced to the BSFL or its feed, (iii) to have used high-throughput amplicon sequencing of the 16S rRNA gene, (iv) to have used a sequence length of at least 250 bp, and (v) to have associated metadata and quality score files. BSFL studies that did not meet all of these criteria are summarized in Table S1. For the 11 studies that met our criteria, the raw sequence data were collected in fastq-format using the NCBI SRA-Toolkit (https://github.com/ncbi/sra-tools). Overall, the studies included BSFL gut samples originating from 13 countries worldwide and whole larvae samples from five European countries. All samples were collected between 2016 and 2021.

**Table 1.** Overview of the raw data collected from each paper. Sample meta-information was collected from the main text as well as from the supplementary meta-data files. The sequencing platform used in all studies used was Illumina Miseq. Position in the 16S rRNA region was determined through an alignment with references from the Silva Living Tree Project Database (v123).

Reference	Assignment to subset(s)	No. of samples	Larval age at harvest	DNA extraction method	Sample type(s)	Country of origin	Data availability (accession no.)	Primers	Alignment 16S rRNA position
Khamis et al. 2020	1	13	No information provided	Isolate II GenomicDNA Kit (Bioline, London, and United Kingdom)	Whole gut	Australia, China, Costa Rica, Ghana, Kenya, Nigeria, South Africa, Thailand, Netherlands, Uganda and United States.	PRJNA625868	LepF1 (5′-ATTCAACCAATCATAAAGATATTGG-3′) and LepR1 (5′-TAAACTTCTGGATGTCCAAAAAATCA-3′)	6388–15969
Liu et al. 2020	1	9	4 to 9 days old larvae	FastDNA Spin Kit for faeces (MP Biomedicals, Illkirch, France)	Whole gut	China	PRJNA639938	314F (5′-CCTAYGGGRBGCASCAG-3′) and 806R (5′-GGACTACNNGGGTATCTAAT-3′)	6332–15885
Tegtmeier et al. 2021a	1	18	L5 instar larvae	NucleoSpin Soil-Kit (Macherey-Nagel Germany)	Whole guts	Germany	PRJNA674583	341F (5′-CCTAYGGGRBGCASCAG-3′) and 806R (5′-GGACTACNNGGGTATCTAAT-3′)	6428–16300
Tegtmeier et al. 2021b	1	6	L5 instar larvae	NucleoSpin Soil-Kit (Macherey-Nagel Germany)	Whole guts	Germany	PRJNA739514	341F (5′-CCTAYGGGRBGCASCAG-3′) and 806R (5′-GGACTACNNGGGTATCTAAT-3′)	6428–16305
Zhan et al. 2020	1	16	8 to 18 days old larvae	Gentra Puregene Yeast/Bact Kit B (Qiagen)	Midguts	China	PRJNA547968	Unknown	6451–15969
Zhang et al. 2020	1	3	20 days old larvae	E.Z.N.A.® Soil Kit (Omega Bio-tek, Norcross, GA, USA)	Whole gut	China	PRJNA612580	338F (5′-ACTCCTACGGGAGGCAGCAG-3′) and 806R (5′-GGACTACHVGGGTWTCTAAT-3′)	6324–15952
Cifuentes et al. 2020	2 and 3	9	Stages S1, S2 and S3	DNeasy Blood & Tissue Kit (Qiagen)	Whole guts	Germany	PRJNA578547	341F (5′-CCTACGGGNGGCWGCAG-3′) and 785R (5′-GACTACHVGGGTATCTAAKCC-3′)	14962–25316
Klammsteiner et al. 2020	2 and 3	35	6 to 27 days old larvae	NucleoSpin R Soil-Kit (Macherey-Nagel Germany)	Whole gut	Germany	PRJEB33904	515F (5′-GTGCCAGCMGCCGCGGTAA-3′) and 806R (5- GGACTACHVGGGTWTCTAAT-3′)	13862–23440
Klammsteiner et al. 2021	2 and 3	36	6 to 26 days old larvae	NucleoSpin R Soil-Kit (Macherey-Nagel Germany)	Whole gut	Austria	PRJEB39545	515F (5′-GTGCCAGCMGCCGCGGTAA-3′) and 806R (5- GGACTACHVGGGTWTCTAAT-3′)	13862–23440
Wynants et al. 2019	3	34	14 to 21 days old larvae	DNeasy Soil Kit (Qiagen, Hilden, Germany)	Whole organism	Belgium, The Netherlands and Switzerland	PRJNA476046	515F (5′-GTGCCAGCMGCCGCGGTAA-3′) and 806R (5′-GGACTACHVGGGTWTCTAAT-3′)	13862–23443
Gorrens et al. 2022	3	84	6 to 25 days old larvae	E.Z.N.A.® Soil Kit (Omega Bio-tek, Norcross, GA, USA)	Whole organism	Belgium, France and Germany	PRJNA742922	515F (5′-GTGCCAGCMGCCGCGGTAA-3′) and 806R (5′-GGACTACHVGGGTWTCTAAT-3′)	13862–23440

### Processing of the 16S rRNA gene sequences

After aligning the sequences against the Silva Living Tree Project reference database v123 (LTP v 123), it became clear that six datasets aligned together (6332–16305), while the other five datasets aligned to a region more downstream in the 16S rRNA gene (13862–25316). As there was only very little overlap between both regions (less than 94 bp in 99% of the alignments), the overall data set was split in three subsets. Subset 1 consisted of six data sets of 16S rRNA sequences from BSFL gut samples spanning the 6388–15969 region in the alignment (corresponding to the V3-V4 region of the 16S rRNA gene). Subset 2 consisted of three data sets containing gut sample sequences covering the 13862–25316 region in the alignment (*i.e*. the V4-V5 region), and Subset 3 consisted of three data sets containing sequences from gut samples and two data sets with sequences from whole larvae samples located at the 13862–25316 region (V4-V5 region) (Yarza et al. 2014) (Table 1). Further analysis of each of the three separate subsets is described below.

Obtained demultiplexed pair-end reads (with primer and barcodes removed) were merged using USEARCH (Edgar 2010) fastq-mergepairs to form consensus sequences with no more than 10 mismatches allowed in the overlap region. Sequences were trimmed at a length of 250 bp with a maximum estimated error of 0.5 (VSEARCH-fastq_filter option (Rognes et al. 2016)). Unique sequences were classified using the Silva Living Tree Project database v123 and non-bacterial sequences (i.e. chloroplasts, mitochondria, Archaea, Eukaryota) were removed using MOTHUR's ‘classify.seqs()’ and ‘remove.lineage()’ commands (Schloss et al. 2009). The resulting decontaminated sequences were classified into zOTUs by the UNOISE3 algorithm as implemented in USEARCH. zOTUs occurring at a relative abundance below a threshold of 0.1% per sample were set to zero. zOTUs were identified using the SINTAX algorithm implemented in USEARCH based on the Silva Living Tree Project database v123. Further, the identity of the most important zOTUs was verified with a BLAST search in GenBank against exemplar species or ‘type materials’ (Federhen 2015). When no significant similarity was found with type materials (<97% identity), the BLAST analysis was performed against entire GenBank. Identification was based on the highest max score, identity percentage and the lowest E-value.

### Data analysis and statistics

#### Core bacteria

Subset 1 and Subset 2 data sets were used to define the ‘core microbiota’ occurring in BSFL guts. Core microorganisms were defined in two ways, first based on a rather stringent definition of zOTUs being present in at least 80% of all samples investigated and secondly based on zOTU presence in more than 50% of all samples. To this end, for each sample, zOTUs were scored as being present or absent in the sample. Presence or absence and relative abundance of the zOTUs over all samples were analysed using the *phyloseq* (McMurdie and Holmes 2013) and *microbiome* (Lahti and Shetty 2012-2019) packages in R 4.0.4 (R Core Team 2021).

#### Factors determining the community composition

For the Subsets 1 and 2, beta diversity estimates (permutational analysis of variance (PERMANOVA) and visualized by non-metric multidimensional scaling (NMDS)) of Hellinger-transformed relative abundance data were used to study the effect of three experimental factors potentially influencing the gut microbiota composition of BSFL, including ‘Study’, ‘Age’ and ‘Feed’. The factor ‘Study’ refers to the fact that each study performed may vary in experimental parameters that may have influenced the microbiota composition (*i.e*. variation in genetic lineages of BSFL used, presence of in-house bacteria, difference in scale at which larvae were reared, difference in DNA-extraction methods, and other possible experimental differences). For the factor ‘Age’, two subgroups were differentiated, including a subgroup ‘Young’ and a subgroup ‘Old’. Distinction between these two subgroups is based on the sample descriptions given in the original studies. Information about the larval age at harvest for each paper is provided in Table 1. The subgroup ‘Young’ consisted of larval samples harvested at an age of 4–14 days, or described as ‘early instar larvae’ in the respective papers. The subgroup ‘Old’ was composed of larval samples harvested at an age of 15 days and above or ‘prepupae’. Larvae indicated with an age of ‘14–17 days’ were categorized as ‘Old’. With regard to the parameter ‘Feed’, samples were grouped into six categories based on the different food sources used. The categories were: ‘Agricultural sidestreams’, ‘Animal feed’, ‘Food/Oil waste’, ‘Fruit/Vegetable waste’, ‘Manure’ and ‘Oily sidestreams’. Larval samples that were reared on substrates that would directly influence the microbiota (such as substrates supplemented with antibiotics or inoculated with microbial strains) were discarded from the analysis, as the microbiota of these samples possibly did not reflect the natural diversity in the samples. Also, six samples reared on poultry blood (Wynants et al. 2019) were removed from the analysis, as this type of rearing substrate is not commonly used in BSFL production. Furthermore, 13 samples originating from the study of Khamis et al. (2020) did not provide any information about larval age or rearing substrate, and they were therefore also discarded from this part of the analysis.

#### Comparison between gut and whole larvae samples

Differences in the microbiota composition between gut and whole larvae samples (Subset 3) were evaluated using the alpha diversity indices zOTU richness, Shannon's diversity index and Simpson's diversity. The student's unpaired t-test (in case of normality and equal variances) and the Wilcoxon Rank Sum test using the Bonferroni's correction (in case of non-normality) were used to reveal any statistically significant differences between both subsets of samples. Further, differences between both subsets were analysed with beta diversity estimates using PERMANOVA and visualized by NMDS, based on a Bray–Curtis matrix of Hellinger-transformed relative abundance data.

## Results

### Meta-analysis characteristics

Data collection resulted in sequences originating from 145 BSFL gut samples to 114 whole BSFL samples, located at different regions of the 16S rRNA gene. These sequences were divided into three subsets, as described earlier based on 16S rRNA gene region (Subset 1 and 2) or sample type (Subset 3). Subset 1 consisted of   7 666  062 sequences originating from 65 BSFL gut samples. Subset 2 consisted of  5 856  794 sequences from 80 BSFL gut samples, and Subset 3 consisted of 8 719 940 sequences representing the same 80 BSFL gut samples combined with 114 whole larvae samples. Subset 1 yielded a total of 6652 zOTUs, of which 281 zOTUs accounted for 95% of the total number of reads. Subset 2 yielded a total of 1736 zOTUs, of which 46 zOTUs accounted for 95% of the total number of reads. Subset 3 contained a total of 3276 zOTUs, of which 154 zOTUs accounted for 95% of the total number of reads. Rarefaction curves for the samples in all three subsets were computed, and suggest that sequencing depth was sufficient to cover microbial diversity (Fig. S1–S3).

### Identification of the core gut bacteria of BSFL

**Figure 1.**
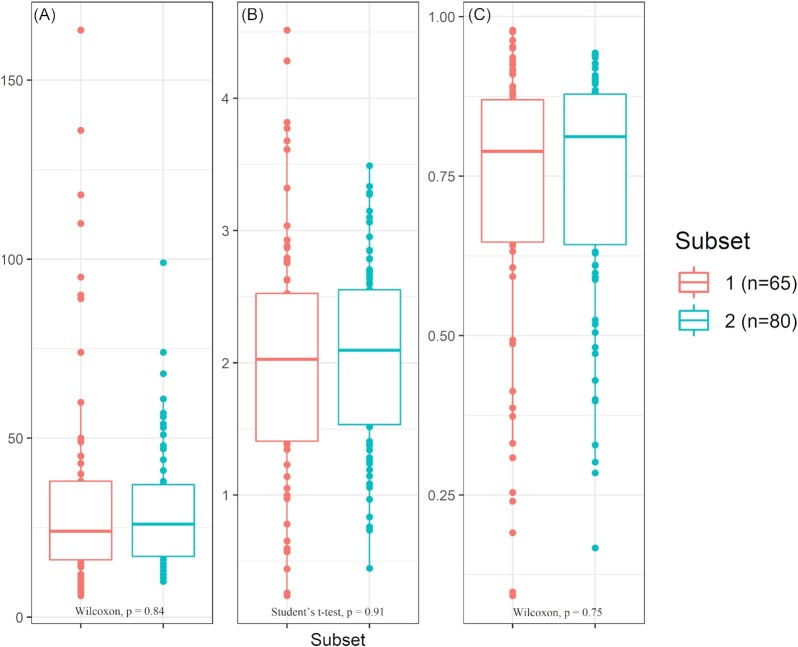
Alpha diversity measurements for BSFL gut samples. Observed zOTU richness **(A)**, Shannon's diversity index **(B)** and Simpson's diversity **(C)** are visualized for BSFL gut samples from Subset 1 (red, n = 65) and Subset 2 (blue, n = 80) using boxplots. Student's unpaired t-tests and Wilcoxon Rank Sum tests are used for significance, *P*-values of these tests are provided under each boxplot.

Core bacteria were identified by analysing both Subset 1 and Subset 2 sequences. Although the number of studies and samples differed between both data sets, evaluation of the alpha diversity revealed no significant differences in the number of observed zOTUs per sample, zOTU richness, nor Shannon's and Simpson's diversity indices between Subsets 1 and 2 (Fig. 1).

**Figure 2.**
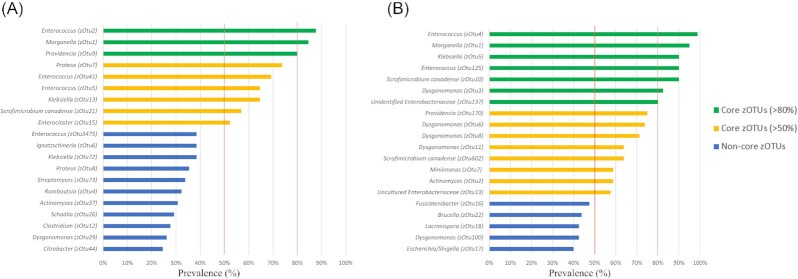
Most prevalent zOTUs in BSFL gut samples in Subsets 1 **(A)** and 2 **(B)**. Prevalence of zOTUs was based on presence and absence of zOTUs across all samples analysed (n = 65 for Subset 1, n = 80 for Subset 2) for each separate subset. zOTUs were identified using BLAST against type materials. When blasting of zOTUs yielded less than 97% sequence identity with type materials, sequences were blasted against entire GenBank. zOTUs were considered to be part of the BSFL core microbiota when they were prevalent in over 80% (green bars) and 50% (yellow bars) of all samples within each group. Blue bars represent prevalent species under these threshold values.

An overview of the identification results of the most prevalent zOTUs from Subsets 1 and 2 is provided in Table S2. When considering the most prevalent bacteria in Subset 1, there were three zOTUs meeting the definition of core microbiota as being present in over 80% of the samples. These included members of *Enterococcus* (zOTU2, present in 87.7% of samples), *Morganella* (zOTU1, 84.6%) and *Providencia* (zOTU9, 80.0%). When applying the less strict definition of core microbiota as being present in at least 50% of the samples, six additional zOTUs were found, representing members of the genera *Proteus* (zOTU7, 73.8%), *Klebsiella* (zOTU13, 64.6%), *Enterococcus* (zOTU5, 64.6%; zOTU41, 69.2%), *Scrofimicrobium* (zOTU21, 56.9%) and *Enterocloster* (zOTU15, 52.3%) (Fig. 2).

In Subset 2, seven bacterial zOTUs were present in more than 80% of the samples investigated, representing members of *Enterococcus* (zOTU4, 98.8%; zOTU125, 90.0%), *Morganella* (zOTU1, 95.0%), *Scrofimicrobium* (zOTU10, 90.0%), *Klebsiella* (zOTU5, 90.0%), *Dysgonomonas* (zOTU3, 82.5%) and an unidentified member of the *Enterobacteriaceae* family (zOTU137, 80.0%). Eight additional zOTUs were included in the core microbiota when the prevalence threshold was lowered to 50% of all gut samples. These additional zOTUs corresponded to species of *Providencia* (zOTU170, 75.0%), *Dysgonomonas* (zOTU6, 73.8%; zOTU8, 71.3%; zOTU11, 63.8%), *Scrofimicrobium* (zOTU602, 63.8%), *Miniimonas* (zOTU7, 58.8%), *Actinomyces* (zOTU2, 58.8%) and an unknown *Enterobacteriaceae* bacterium (zOTU13, 57.5%) (Fig. 2).

### Factors affecting the bacterial composition of BSFL gut

**Figure 3.**
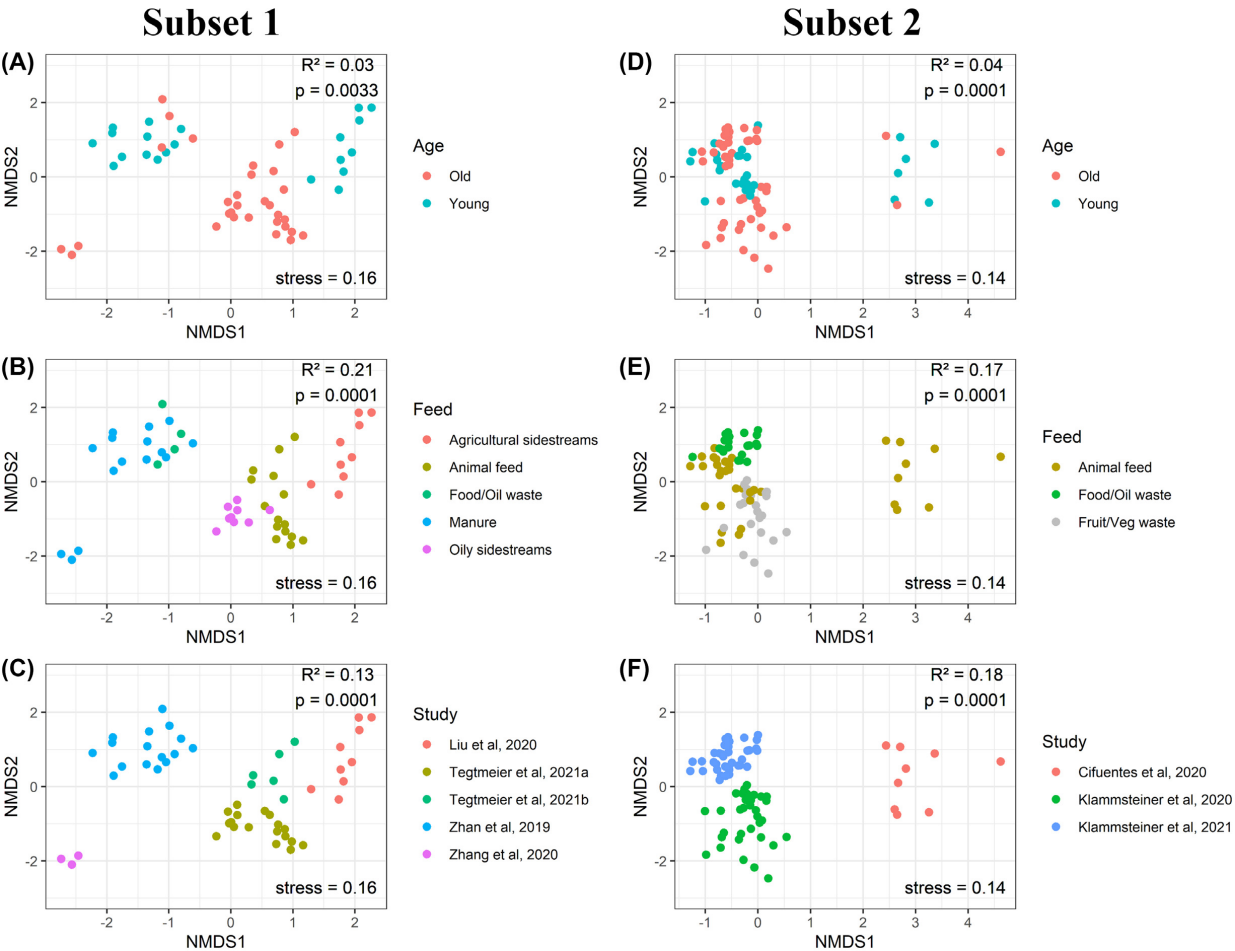
NMDS plots showing clustering of larval gut samples based on Bray-Curtis matrices of Hellinger-transformed relative abundance data. Plots show clustering of gut samples of Subset 1 **(A, B, C)**and Subset 2 **(D, E, F)** for three different factors: ‘Age’, ‘Feed’ and ‘Study’. Results of PERMANOVA are shown in the upper right corner of each plot and reveal the significance (*P*-value) of each factor and the amount of variation that the factor explains (R^2^). Stress values of the NMDS ordination are shown in the lower right corner of each plot.

NMDS clustering of the data from Subsets 1 and 2 revealed clustering of samples based on ‘Age’ for both subsets (Fig. 3A and 3D). Clustering based on ‘Feed’ can be observed in Figs 3B and 3E, respectively, for Subset 1, and based on ‘Study’ in Figs 3C and 3F, respectively, for Subset 2. PERMANOVA of the Hellinger transformed Bray-Curtis dissimilarities revealed that each of these three factors had a significant effect on the total bacterial community composition found in the gut samples of both subsets, and when they were combined, they explained nearly 40% of all the variation (Table 2). In Subset 1, the factor ‘Feed’ explained most of the variation between the samples, followed by ‘Study’. The factor ‘Age’ only accounted for a small proportion of the variation among samples. For Subset 2, ‘Study’ and ‘Feed’ also accounted for a larger proportion of the variation relative to ‘Age’.

**Table 2.** Results of PERMANOVA analysis of factors affecting the gut microbiota of BSFL. Tested factors had a significant effect on the microbiota composition found in gut samples of Subsets 1 and 2. Statistical testing of Bray-Curtis dissimilarities of Hellinger-transformed relative abundance data was done using PERMANOVA with the adonis function in R with 9999 permutations.

	Subset 1		Subset 2	
Factor	R^2^	*P*-value	R^2^	*P*-value
*Age*	0.03	0.0033	0.04	0.0001
*Feed*	0.21	0.0001	0.17	0.0001
*Study*	0.13	0.001	0.18	0.0001

### Difference in bacterial composition between gut and whole larval samples

**Figure 4.**
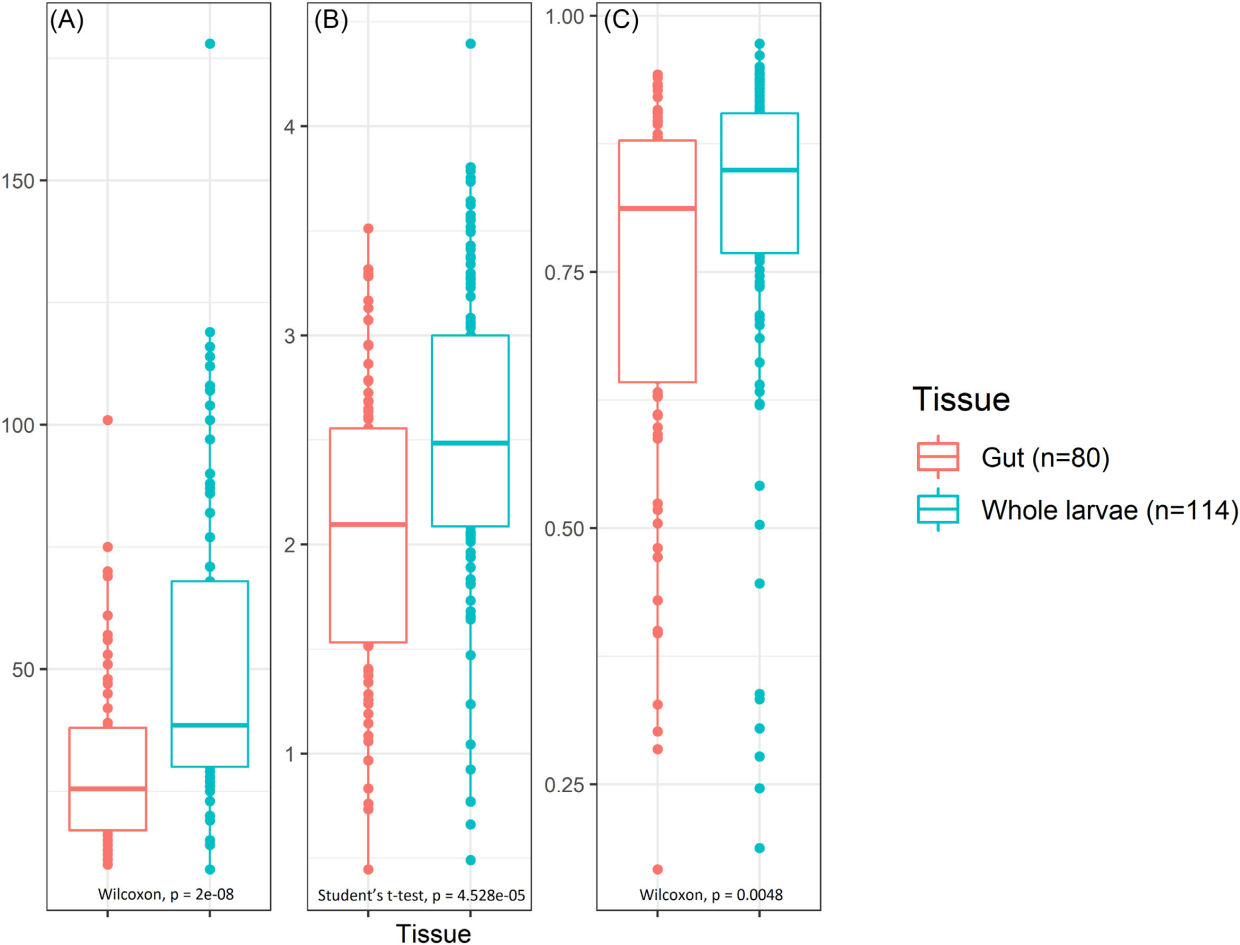
Alpha diversity measurements for gut and whole larvae samples from Subset 3. Observed zOTU richness **(A)**, Shannon's diversity index **(B)** and Simpson's diversity **(C)** are visualized for gut (red, n = 80) samples and whole larvae (blue, n = 114) samples using boxplots. Student's unpaired t-tests and Wilcoxon Rank Sum tests are used for significance, *P*-values of these tests are provided under each boxplot.

**Figure 5.**
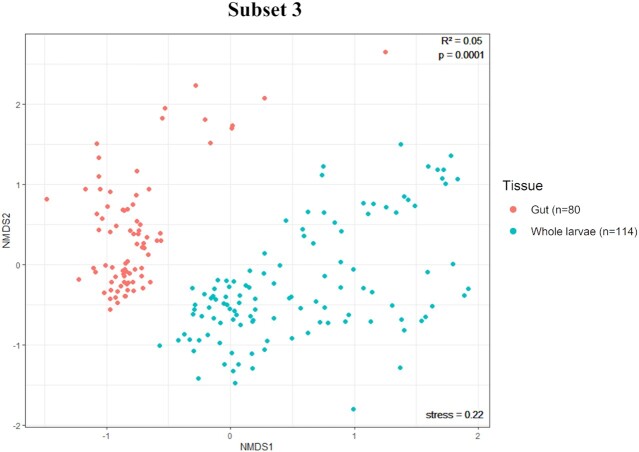
NMDS plot of Bray-Curtis dissimilarities of Hellinger transformed relative abundance data of Subset 3. Grouping occurred based on the factor ‘Tissue’ and distinguishes gut (red, n = 80) and whole larvae (blue, n = 114) samples. Results of PERMANOVA are shown in the upper right corner of the plot and reveal the significance (*P*-value) of the factor ‘Tissue’ and the amount of variation that the factor explains (R^2^). The stress value of the NMDS ordination is shown in the lower right corner of each plot.

Alpha diversity determinations of Subset 3 revealed that the observed number of zOTUs and bacterial diversity was significantly higher in the ‘Whole larvae’ samples than in the ‘Gut’ samples, as illustrated in Fig. 4. A beta-analysis NMDS-plot of Bray-Curtis values (Fig. 5) showed clear grouping based on the factor ‘Tissue’ (representing either ‘gut samples’ or ‘whole larvae samples’). Furthermore, PERMANOVA results showed that the factor ‘Tissue’ had a significant (*P* = 0.0001) effect, although a relatively low share in the variation was explained by this factor (R^2^ = 0.05) (Table 3).

**Table 3.** Results of PERMANOVA analysis of the factor ‘Tissue’ on BSFL microbiota composition. The factor ‘Tissue’ had a significant effect on the microbiota composition found in ‘Gut’ and ‘Whole larvae’ samples within Subsets 3. Statistical testing of Bray-Curtis dissimilarities of Hellinger-transformed relative abundance data was done using PERMANOVA with the adonis function in R with 9999 permutations.

	Subset 3	
Factor	R^2^	*P*-value
*Tissue*	0.05	0.0001

## Discussion

### Core bacterial genera reoccur in BSFL gut samples despite inter-study variations

Despite the variations among datasets, several bacterial genera showed a high prevalence in both BSFL gut sample subsets analysed. zOTUs representing members of *Enterococcus* and *Morganella* were found in over 80% of the samples and were therefore included in our most strict definition of the core bacteria of BSFL. zOTUs identified as members of the genus *Providencia* were found in more than 75% of all samples in both subsets. The less strict definition of core microbiota also included members of *Scrofimicrobium* and *Klebsiella* in both gut sample subsets. With the exception of *Scrofimicrobium*, members of these genera have also been identified in other BSFL studies which are not included in this meta-analysis (Ao et al. 2020, Gold et al. 2021). *Scrofimicrobium* is a new genus of the *Actinomycetaceae* family, which is closely related to *Actinomyces* and only described so far by Wylensek et al. (2020) based on isolates from the pig gut. *Actinomyces* has been found before in BSFL gut studies (Ao et al. 2020, Galassi et al. 2021, Gold et al. 2021). Based on this meta-analysis, members of the above mentioned bacteria can be considered core genera of the BSFL gut community. However, this does not exclude other bacterial genera to belong to the core microbiota of the BSFL gut. Indeed, increasing the number of samples in future meta-analyses can provide additional insights in omnipresent bacterial genera in the BSFL gut.

Future research can be dedicated to unravel the function(s) of BSFL core gut bacteria, which is still a largely unknown field. However, knowledge of the functionality of these bacteria in other (insect) species might provide useful insights in the functions these microorganisms could have in BSFL. For example, enterococci are found in a wide variety of ecological niches, such as the gastro-intestinal (GI) tracts of human, animal and insect hosts, but also in plants, soil, water, fermented foods and dairy products (Lebreton et al. 2014). Although many *Enterococcus* strains are considered pathogenic (Murray, 1990), Shao et al. (2017) found that in the Egyptian cotton leafworm (*Spodoptera littoralis*), the native gut bacterium *Enterococcus mundtii* has an inhibiting effect on pathogenic enterococcal bacteria such as *Enterococcus faecalis* and other gram-positive bacteria by altering the environment of the host and thereby increasing host survival (Shoa et al., 2017). Possibly the *Enteroccocus* strains found in the BSFL gut have similar antagonistic effects. Another possible function of *Enterococcus* in BSFL was proposed by Yang et al. (2021), where it was hypothesised that certain *Enterococcus* species might influence host hunger and trigger increased feeding behaviours. *Morganella morganii* has been reported to cause mortality in the Mexican fruit fly (*Anastrepha ludens*) (Salas et al. 2017). However, the species is also observed to produce phenol, which serves as a sex pheromone in the New Zealand grass grub (*Costelytra zealandica*) (Marshall et al. 2016). Although the function of *Morganella* in BSFL is currently undocumented, some members of *Morganella* and *Providencia* have a role in urea hydrolysis (Gold et al. 2020), while addition of urea or ammonia to BSFL diet has led to increased larval biomass (Ren et al. 2022). Further research is needed to find out whether these metabolic benefits are facilitated by BSFL gut bacteria. *Providencia* and *Ignatzschineria* have also been found to affect oviposition behaviour of adult black soldier flies. Black soldier flies avoid sites inoculated with *Ignatzschineria* strains isolated from eggs of competing fly species. Most likely the flies use the bacterial odours to find suitable oviposition sites, to minimize competition with others for the same resources (Zheng et al. 2013b). However, black soldier flies positively responded to a *Providencia* strain isolated from another fly species, maybe because the species utilizes disparate resources (Zheng et al. 2013b). In BSFL, it is suggested that *Ignatzschineria*, *Providencia* and *Enterococcus* are potential hosts of genes that could aid the degradation of oxytetracycline, which would benefit the ability of the larvae to feed on waste streams containing high amounts of this antibiotic (Liu et al. 2020). Furthermore, *Providencia* has been proposed to play a role in protein and lipid conversion in the BSFL gut (Ao et al. 2020) and to produce xylanases (Raj et al. 2013), which can assist the insect with the digestion of plant cell walls (Sontowski and van Dam 2020). However, several members of this genus have also shown to be pathogenic to fruit flies (*Drosophila melanogaster*) (Galac and Lazzaro 2011). Degradation of plant material and chitin has also been achieved through enzymatic activity of *Actinomyces*, which can aid BSFL in reaching their nutritional requirements (Yang et al. 2021). Also, *Actinomyces olivocinereus* has been reported to produce the antibiotic heliomycin, which has antibacterial and mycobacterial properties (Sharma et al. 2014). Possibly, the closely related *Scrofimicrobium* genus found in the BSFL microbiota provides similar metabolic or protective functions for the larvae, but this should still be confirmed. *Klebsiella*, alongside several other gut bacteria such as *Enterobacter* and *Pseudomonas*, has been reported to produce antioxidants that reduce the amount of toxic oxidants in blood-feeding insects (Sontowski and van Dam 2020). In BSFL pectinolytic activity of *Klebsiella* has been reported (Gorrens et al. 2021). Furthermore, the high relative abundance of *Klebsiella* found in BSFL reared on cellulose- and hemi-cellulose rich substrates suggests that this bacterium might be involved in the catabolism of such compounds (Gorrens et al. 2021). The presence of such bacteria that help in digestion or in neutralizing toxic components might explain why BSFL are able to survive and grow on a wide diversity of substrates. Unravelling the function of BSFL core bacteria can provide more insight in the relation between the insect and its microbiota, as well as in its consequences for insect performance and the commercial production of BSFL.

### Experimental parameters affecting the gut bacterial composition

The results of this meta-analysis show that experimental parameters such as larval age at harvest or rearing substrate influence the BSFL gut microbiota composition. This was shown in some earlier studies as well (Zheng et al. 2013a, Bruno et al. 2019, Ao et al. 2020, Cifuentes et al. 2020, Osimani et al.^2021^), but the added value of a meta-analysis is the possibility to assess whether or to what extent results depend on specific studies. Although some types of substrate were placed in the same category for this analysis, variation between feeds within one category may still be present. For example, chicken feed was often used as a control feed, but the composition of the chicken feed likely varied over different studies. Consequently, its nutritional value and microbial load differed between the studies, which in turn may have been reflected in the microbial composition of the larvae. Similarly, the factor ‘Age’ only differentiated between ‘Young’ and ‘Old’ larvae to make a comparison between both groups, yet the microbiota composition of larvae within these categories can still vary. Because not all studies use the same type of feed and the same larval age at harvest, not all variation can be ascribed solely to these factors. The gut microbiota is more likely influenced by a range of factors and their interactions. Within the parameter ‘Study’, some factors that potentially influence the microbiota composition might not be captured in this analysis. A relatively large proportion of the microbiota variation is explained by this combination of unknown factors summarized in the factor ‘Study’, which has also been observed in cattle microbiota studies (Holman et al. 2017, Holman and Gzyl 2019). One of these factors might be the in-house microbiota of rearing facilities used in certain studies.

An example of the fact that in-house microbes can impact the composition of the gut microbiota is the high prevalence of *Dysgonomonas* species in Subset 2. The four zOTUs associated with *Dysgonomonas* are present in more than 50% of the samples included in Subset 2, whereas *Dysgonomonas* is significantly less prevalent in samples from Subset 1. When zooming in on subset 2, it is found that 99.98% of the sequence reads of these four *Dysgonomonas* zOTUs originate from only two studies by Klammsteiner et al. (2020, 2021). While these studies do originate from the same facility, they used a range of different feed types. Nevertheless, the abundance of the most prominent *Dysgonomonas* zOTU (zOTU3) remained similar regardless of the studied feed type (Fig. S4). This also suggests that this zOTU is more likely member of the in-house flora of the rearing facility, rather than a typical member of the BSFL core species. Other factors such as genetic variation in BSFL have not been included in this analysis and it is not known yet whether they should be taken into consideration when evaluating the effect of experimental parameters.

Although core bacteria were found in diverse samples, their relative abundance was found to vary substantially. For example, zOTUs identified as *Morganella* in Subset 1 accounted on average for 7.0% of all reads, but their relative abundance varied greatly among samples: zOTUs identified as *Morganella* covered between 0.16% and 95.2% of the reads in a sample (Fig. S5). This pattern occurred for other core bacteria as well, as the number of reads varied between a minimum of 0–1% and a maximum of 61.1% (*Enterococcus*), 75.9% (*Providencia*), 15.8% (*Klebsiella*) and 3.9% (*Scrofimicrobium*). For Subset 2, similar observations can be made (Fig. S6). These observed variations in number of reads can be attributed to some extent to the specific experimental factors. This can be illustrated by considering the individual samples from the Tegtmeier et al. (2021a) dataset analysed in Subset 1 (Fig. S5). These BSFL samples all originated from the same insect producer, were used in the same study and were harvested at the same larval instar phase. The only experimental variable in this study was ‘Feed’, where half of the samples were reared on control chicken feed and the other half on cottonseed press cake (CPC). zOTUs identified as *Morganella* were present in all samples, but the relative abundance was significantly higher in the control feed (on average 75.8%) compared to CPC feed (on average 21.7%), indicating that ‘Feed’ strongly influenced the relative abundance of *Morganella*. However, not all variation in relative abundances can be explained by this factor. There was also variation between samples within the same feeding group, where the relative abundance of *Morganella* was very high in some samples of the CPC group and very low in one of the samples from the control group. This variation in relative abundance, which was also noticeable for other core bacteria, is unlikely related to the experimental conditions (such as relative humidity, rearing conditions, crowding, age, and feed), since these were equal for each sample. These examples illustrate the plasticity of the core bacteria: they are prevalent in most samples, but their relative abundance can clearly vary and the exact triggers behind these changes remain to be determined.

### Challenges in performing a meta-analysis of the BSFL microbiota

In any meta-analysis, the aim is to obtain the highest number of similar samples in order to limit the amount of variation and draw general conclusions about the effects of the experimental variables. Data availability and clear sample description is therefore of utmost importance for any meta-analysis. In BSFL next generation sequencing research, there are studies that sequence (part of) the dissected larval gut and studies that use the whole, undissected larvae. In this study, it was shown that there is variation in bacterial species diversity between ‘Gut’ and ‘Whole larvae’ samples. The bacterial species diversity was higher when whole larvae were examined compared to the larval gut. The higher number of species observed in the whole larvae might be caused by environmental bacteria that reside on the surface of the insect or enter through wounds in the cuticle (Joosten et al. 2020). Future research should carefully consider these variations and involve the appropriate sample material based on research questions and design. Variation in the microbiota composition between different parts of the insect has been demonstrated before. Deguenon et al. (2019) found a significant higher bacterial diversity on the external surface of the black blow fly (*Phormia regina*) compared to the gut of the fly. A higher bacterial diversity in external samples has also been found in the house fly (*Musca domestica*) (Park et al. 2019) and in both healthy and parasitized cabbage white (*Pieris brassicae*) caterpillars (Gloder et al. 2021). However, it must be noted that in these studies, the external microbiota was obtained by washing the insect cuticle and thus did not involve any insect tissue. Nonetheless, due to this kind of variation, it is important to draw separate conclusions regarding the ‘Gut’ microbiota and a more general ‘Whole Larvae’ microbiota. This should be reflected in the terminology used in scientific papers. Research on both the ‘Gut microbiota’ (using dissected samples) and the ‘Whole microbiota’ (using whole larvae) is useful, but each in a different context. Focusing on the ‘Gut microbiota’ is relevant in fundamental studies on the composition and functions of gut micro-organisms. Information on the ‘Gut microbiota’ expands the knowledge on conditions enabling efficient digestion of (sometimes difficult-to-digest) substrates (such as some organic waste streams), and hence growth and yield of BSFL production. Considering the ‘Whole microbiota’ is a good approach when studying the microbiological food safety and shelf life of BSFL, or other quality aspects in industrial production. Furthermore, since it does not require dissection, analysis of whole larvae samples is less time consuming, which allows for greater sample sizes in these kind of experiments.

In either gut or whole larvae next generation sequencing, the targeted region of the 16S rRNA gene, primer choices, DNA extraction methods and bio-informatical parameters vary among studies. As a result, the data used in this meta-analysis originated from two different genetic regions, with very little overlap between them and resulting in two separate analyses. This variation in sequenced genetic region can impact the observed microbiota composition, as observed in the swine gut by Holman et al. (2017), who studied the effect of this parameter on the microbiota composition in the swine gut. When comparing results derived from the V1-V3 and V4 region of the 16S rRNA gene, they found a higher Simpson's reciprocal value in samples from the V4 region, although there was no variation found in species richness and Shannon index data (Holman et al. 2017). Holman et al. (2017) further noticed that on the genus level some bacteria were far less abundant when sequences originated from the V1-V3 region compared to the V4 region. In this study by Holman et al. (2017), the choice of the selected 16S rRNA gene variable region might have resulted in an underestimation of relative abundances of these bacterial genera. In the current study, there were no significant differences in species diversity between gut samples from Subset 1 and Subset 2. Although the results of both analyses are therefore comparable, potential underestimation of the number of bacterial genera in either Subset 1 or 2 due to the region selection might still be possible. Future meta-analyses would benefit from uniformity among researchers in DNA extraction methods, primer choices and bioinformatics pipeline usage. The usage of an identical set of primers across different studies such as the 515F (5'—GTG CCA GCM GCC GCG GTA A–3') and 806R (5'—GGA CTA CHV GGG TWT CTA AT–3') (Caporaso et al. 2011) and limiting the length of processed reads to 250 bp would result in more comparable results among studies. Usage of a universal bioinformatics pipeline among microbiota researchers might be unlikely, since some software can be more suitable for specific tasks such as data visualization or statistics and the usage of such programs is often a personal preference of the researcher. Nevertheless, although the analytical methods are often described to some degree, a standardized method of reporting the bioinformatics process could increase the repeatability and robustness of bioinformatic analyses. This includes information about software used (including version numbers), sequence length and error thresholds, methods of data filtering, as well as referenced databanks, which all could be added in a supplementary table to the manuscript.

Variation in data generation and analysis is not the only pitfall of BSFL microbiota analysis. Interpretation of results can be influenced by the use of different definitions, *e.g*.of the ‘core’ microbiota. In this study, the intrinsic core bacterial community of BSFL was defined as those bacteria that were found above a prevalence threshold of either 80% or 50% across all samples studied, regardless of external factors (*i.e*. experimental setup, larval age, rearing facility). This definition follows the same criteria as the ‘Common core’ definition by Risely (2020) and it can be quantified by scoring the presence or absence of bacteria found in samples, as described by Shade and Handelsman (2012). This method, however, does not take into account the relative abundance of bacterial taxa found in the samples (Shade and Handelsman 2012), nor does it provide any information about the function of the bacterial species or their dynamics over time (Risely 2020). The definition of ‘core’ bacterial species varies greatly among (black soldier fly) studies: the core microbiota can be described as bacterial groups present in all samples regardless of environmental and/or experimental conditions (Ao et al. 2020), as groups being abundant in a certain percentage of samples (Klammsteiner et al. 2020, Shelomi et al. 2020) or as groups persistent over life stages and time (Cifuentes et al. 2020, Raimondi et al. 2020, Zhan et al. 2020). Several definitions can exist next to each other, as long as studies clearly describe the definition(s) they consider, so that comparisons between studies can be made correctly.

## Conclusions

This study evaluated the effects of experimental parameters and bio-informatical choices on the bacterial community composition of BSFL, using data originating from dissected guts as well as whole larvae. Results revealed variation in species diversity between the sample types. Hence, including only the gut or the whole larvae is important to consider when designing future BSFL microbiota experiments, and the choice should be aligned with the aim of the study. Through the meta-analysis, core gut bacterial genera were identified. Regardless of encountered variations, members of the genera *Enterococcus, Morganella* and *Providencia* were the most prevalent in BSFL gut samples, followed by *Klebsiella* and *Scrofimicrobium*. Furthermore, there is room for improvement in standardizing DNA extraction methods and 16S rRNA gene sequence processing, so that more BSFL sequencing data can be combined and analysed in future research. This will allow more profound insights and eventually practical recommendations for BSFL production on a wide scale.

## Supplementary Material

fiac094_Supplemental_Files

## References

[bib1] Albertsen M , Karst SM, Ziegler AS et al. Back to basics–the influence of DNA extraction and primer choice on phylogenetic analysis of activated sludge communities. PLoS One. 2015;10:e0132783. 26182345 10.1371/journal.pone.0132783 PMC4504704

[bib2] Ao Y , Yang C, Wang S et al. Characteristics and nutrient function of intestinal bacterial communities in black soldier fly (*Hermetia illucens* L.) larvae in livestock manure conversion. Microb Biotechnol. 2020;14:886–96. 32449587 10.1111/1751-7915.13595 PMC8085981

[bib3] Aronson AI , Beckman W, Dunn P. *Bacillus thuringiensis* and related insect pathogens. Microbiol Rev. 1986;50:1–24. 3007957 10.1128/mr.50.1.1-24.1986 PMC373051

[bib4] Belghit I , Liland NS, Gjesdal P et al. Black soldier fly larvae meal can replace fish meal in diets of sea-water phase Atlantic salmon (*Salmo salar*). Aquaculture. 2019;503:609–19.

[bib5] Bruno D , Bonelli M, De Filippis F et al. The intestinal microbiota of *Hermetia**illucens* larvae is affected by diet and shows a diverse composition in the different midgut regions. Appl Environ Microbiol. 2019;85:e01864–18. 30504212 10.1128/AEM.01864-18 PMC6328772

[bib6] Caligiani A , Marseglia A, Leni G et al. Composition of black soldier fly Prepupae and systematic approaches for extraction and fractionation of proteins, lipids and chitin. Food Res Int. 2018;105:812–20. 29433277 10.1016/j.foodres.2017.12.012

[bib71_966_220222] Callahan BJ, McMurdie PJand Holmes SP. Exact sequence variants should replace operational taxonomic units in marker-gene data analysis. ISME J. 2017;11:2639–43. 28731476 10.1038/ismej.2017.119 PMC5702726

[bib7] Caporaso JG , Lauber CL, Walters WA et al. Global patterns of 16S rRNA diversity at a depth of millions of sequences per sample. Proc Natl Acad Sci. 2011;108:4516–22. 20534432 10.1073/pnas.1000080107 PMC3063599

[bib8] Cifuentes Y , Glaeser SP, Mvie J et al. The gut and feed residue microbiota changing during the rearing of *Hermet**ia**illucens* larvae. Antonie Van Leeuwenhoek. 2020;113:1323–44. 32638136 10.1007/s10482-020-01443-0

[bib9] Deguenon JM , Travanty N, Zhu J et al. Exogenous and endogenous microbiomes of wild-caught *phormia**regina* (Diptera: Calliphoridae) flies from a suburban farm by 16S rRNA gene sequencing. Sci Rep. 2019;9:1–13. 31889104 10.1038/s41598-019-56733-z PMC6937299

[bib10] Diener S , Zurbrügg C, Tockner K. Conversion of organic material by black soldier fly larvae: establishing optimal feeding rates. Waste Manag Res. 2009;27:603–10. 19502252 10.1177/0734242X09103838

[bib11] Edgar RC . Search and clustering orders of magnitude faster than BLAST, Bioinformatics. 2010;26:2460–1. 20709691 10.1093/bioinformatics/btq461

[bib72_1661159822398] Edgar RC . UNOISE2: improved error-correction for Illumina 16S and ITS amplicon sequencing. BioRxiv. 2016;081257.

[bib12] Engel P , Moran NA. The gut microbiota of insects–diversity in structure and function. FEMS Microbiol Rev. 2013;37:699–735. 23692388 10.1111/1574-6976.12025

[bib13] Federhen S . Type material in the NCBI taxonomy database. Nucleic Acids Res. 2015;43:D1086–98. 25398905 10.1093/nar/gku1127 PMC4383940

[bib14] Fouhy F , Clooney AG, Stanton C et al. 16S rRNA gene sequencing of mock microbial populations-impact of DNA extraction method, primer choice and sequencing platform. BMC Microbiol. 2016;16:1–13. 27342980 10.1186/s12866-016-0738-z PMC4921037

[bib15] Galac MR , Lazzaro BP. Comparative pathology of bacteria in the genus *providencia* to a natural host, *Drosophila**melanogaster*. Microbes Infect. 2011;13:673–83. 21354324 10.1016/j.micinf.2011.02.005 PMC3109104

[bib16] Galassi G , Jucker C, Parma P et al. Impact of agro-industrial byproducts on bioconversion, chemical composition, in vitro digestibility, and microbiota of the black soldier fly (Diptera: Stratiomyidae) larvae. Journal of Insect Science. 2021;21:8. 10.1093/jisesa/ieaa148 PMC7820701 33480429

[bib17] Gloder G , Bourne ME, Verreth C et al. Parasitism by endoparasitoid wasps alters the internal but not the external microbiome in host caterpillars. Animal Microbiome. 2021;3:1–15. 34654483 10.1186/s42523-021-00135-y PMC8520287

[bib19] Gold M , Fowles T, Fernandez-Bayo JD et al. Effects of rearing system and microbial inoculation on black soldier fly larvae growth and microbiota when reared on agri-food by-products. J Insects Food Feed. 2021;1–16.

[bib18] Gold M , Von Allmen F, Zurbrügg C et al. Identification of bacteria in two food waste black soldier fly larvae rearing residues. Front Microbiol. 2020;11:2897. 10.3389/fmicb.2020.582867 PMC7719680 33329446

[bib21] Gorrens E , De Smet J, Vandeweyer D et al. The bacterial communities of black soldier fly larvae (*Hermetia illucens*) during consecutive, industrial rearing cycles. J Insects Food Feed. 2022;1–16.

[bib20] Gorrens E , Van Moll L, Frooninckx L et al. Isolation and identification of dominant bacteria from black soldier fly larvae (*Hermetia illucens*) envisaging practical applications. Front Microbiol. 2021;12:1110. 10.3389/fmicb.2021.665546 PMC8155639 34054771

[bib22] Hiergeist A , Gläsner J, Reischl U et al. Analyses of intestinal microbiota: culture versus sequencing. ILAR J. 2015;56:228–40. 26323632 10.1093/ilar/ilv017

[bib23] Holman DB , Brunelle BW, Trachsel J et al. Meta-analysis to define a core microbiota in the swine gut. MSystems. 2017;2:e00004–17. 28567446 10.1128/mSystems.00004-17 PMC5443231

[bib24] Holman DB , Gzyl KE. A meta-analysis of the bovine gastrointestinal tract microbiota. FEMS Microbiol Ecol, 2019;95:fiz072. 31116403 10.1093/femsec/fiz072

[bib25] Huang Y , Yu Y, Zhan S et al. Dual oxidase gene duox and Toll-like receptor 3 gene TLR3 in the toll pathway suppress zoonotic pathogens through regulating the intestinal bacterial community homeostasis in *Hermetia**illucens* L. PLoS One. 2020; 15:e0225873. 32352968 10.1371/journal.pone.0225873 PMC7192390

[bib26] Joosten L , Lecocq A, Jensen AB et al. Review of insect pathogen risks for the black soldier fly (*Hermetia illucens*) and guidelines for reliable production. Entomol Exp Appl. 2020;168:432–47.

[bib27] Khamis FM , Ombura FL, Akutse KS et al. Insights in the global genetics and gut microbiome of black soldier fly, *Hermetia**illucens*: implications for animal feed safety control. Front Microbiol. 2020;11:1538. 32774330 10.3389/fmicb.2020.01538 PMC7381391

[bib29] Klammsteiner T , Walter A, Bogataj T et al. Impact of processed food (canteen and oil wastes) on the development of black soldier fly (*Hermetia illucens*) larvae and their gut microbiome functions. Front Microbiol. 2021;12:20. 10.3389/fmicb.2021.619112 PMC7858275 33552039

[bib28] Klammsteiner T , Walter A, Bogataj T et al. The core gut microbiome of black soldier fly (*Hermetia illucens*) larvae raised on low-bioburden diets. Front Microbiol. 2020;11:993. 32508795 10.3389/fmicb.2020.00993 PMC7253588

[bib30] Lahti L , Shetty S. 2012-2019. “microbiome R package.” 31719224

[bib31] Lebreton F , Willems RJL, Gilmore MS. Enterococcus diversity, origins in nature, and gut colonization. In: Gilmore MS, Clewell DB, Ike Y, Shankar N. (eds) Enterococci: from commensals to leading causes of drug resistant infection [Internet]. Boston:Massachusetts Eye and Ear Infirmary 2014. PMID: 24649513. 24649513

[bib33] Liu C , Yao H, Chapman SJ et al. Changes in gut bacterial communities and the incidence of antibiotic resistance genes during degradation of antibiotics by black soldier fly larvae. Environ Int. 2020;142:105834. 32540627 10.1016/j.envint.2020.105834

[bib32] Makkar HP , Tran G, Heuzé V et al. State-of-the-art on use of insects as animal feed. Anim Feed Sci Technol. 2014;197:1–33.

[bib34] Marshall DG , Jackson TA, Unelius CR et al. *Morganella morganii* bacteria produces phenol as the sex pheromone of the New Zealand grass grub from tyrosine in the Colleterial gland. Sci Nat. 2016;103:1–6. 10.1007/s00114-016-1380-1 27352077

[bib36] McMurdie PJ , Holmes S. “phyloseq: an r package for reproducible interactive analysis and graphics of microbiome census data.” PLoS One. 2013;8:e61217. 23630581 10.1371/journal.pone.0061217 PMC3632530

[bib35] Miranda CD , Cammack JA, Tomberlin JK. Mass production of the black soldier fly, *Hermetia**illucens* (L.),(Diptera: Stratiomyidae) reared on three manure types. Animals. 2020;10:1243. 10.3390/ani10071243 PMC7401607 32708338

[bib73_819_222122] Murray BE . The life and times of the Enterococcus. Clin Microbiol Rev. 1990;3:46–65. 2404568 10.1128/cmr.3.1.46 PMC358140

[bib37] O'Hara E , Neves AL, Song Y et al. The role of the gut microbiome in cattle production and health: driver or passenger? Ann Rev Anim Biosci. 2020;8:199–220. 32069435 10.1146/annurev-animal-021419-083952

[bib38] Osimani A , Ferrocino L, Corvaglia M et al. Microbial dynamics in rearing trials of Hermetia illucens larvae fed coffee silverskin and microalgae. Food Research International. 2021;140:110028. 33648256 10.1016/j.foodres.2020.110028

[bib39] Oulas A , Pavloudi C, Polymenakou P et al. Metagenomics: tools and insights for analyzing next-generation sequencing data derived from biodiversity studies. Bioinf Biol Insights. 2015;9:BBI–S12462. 10.4137/BBI.S12462 PMC4426941 25983555

[bib40] Park R , Dzialo MC, Spaepen S et al. Microbial communities of the house fly *Musca**domestica* vary with geographical location and habitat. Microbiome. 2019;7:1–12. 31699144 10.1186/s40168-019-0748-9 PMC6839111

[bib41] R Core Team . R: A language and environment for statistical computing. R Foundation for Statistical Computing, Vienna, Austria, 2021. URLhttps://www.R-project.org/.

[bib42] Raimondi S , Spampinato G, Macavei LI et al. Effect of rearing temperature on growth and microbiota composition of *Hermetia**illucens*. Microorganisms. 2020;8:902. 10.3390/microorganisms8060902 PMC7355568 32549385

[bib43] Raj A , Kumar S, Singh SK et al. Characterization of a new *providencia**sp*. strain X1 producing multiple xylanases on wheat bran. Scient World J. 2013;2013. 10.1155/2013/386769 PMC3856158 24348154

[bib44] Ren X , Guo R, Akami M et al. Nitrogen acquisition strategies mediated by insect symbionts: a review of their mechanisms, methodologies, and case studies. Insects. 2022;13:84. 35055927 10.3390/insects13010084 PMC8781418

[bib45] Rintala A , Pietilä S, Munukka E et al. Gut microbiota analysis results are highly dependent on the 16S rRNA gene target region, whereas the impact of DNA extraction is minor. J Biomol Techniq: JBT. 2017;28:19. 10.7171/jbt.17-2801-003 PMC5330390 28260999

[bib46] Risely A . Applying the core microbiome to understand host–microbe systems. J Anim Ecol. 2020;89:1549–58. 32248522 10.1111/1365-2656.13229

[bib47] Rognes T , Flouri T, Nichols B et al. VSEARCH: a versatile open source tool for metagenomics. PeerJ. 2016;4:e2584. 27781170 10.7717/peerj.2584 PMC5075697

[bib48] Salas B , Conway HE, Schuenzel EL et al. *Morganella morganii* (Enterobacteriales: Enterobacteriaceae) is a lethal pathogen of mexican fruit fly (Diptera: Tephritidae) larvae. Florida Entomologist. 2017;100:743–51.

[bib50] Schloss PD , Westcott SL, Ryabin T et al. Introducing mothur: open-source, platform-independent, community-supported software for describing and comparing microbial communities. Appl Environ Microbiol. 2009;75:7537–41. 19801464 10.1128/AEM.01541-09 PMC2786419

[bib49] Shade A , Handelsman J. Beyond the venn diagram: the hunt for a core microbiome. Environ Microbiol. 2012;14:4–12. 22004523 10.1111/j.1462-2920.2011.02585.x

[bib74_143_223022] Shao Y, Chen B, Sun C. Symbiont-Derived Antimicrobials Contribute to the Control of the Lepidopteran Gut Microbiota. Cell Chem Biol. 2017;24:66–75. 28107652 10.1016/j.chembiol.2016.11.015

[bib51] Sharma M , Dangi P, Choudhary M. Actinomycetes: source, identification, and their applications. Internat J Curr Microbiol Appl Sci. 2014;3:801–32.

[bib52] Shelomi M , Wu MK, Chen SM et al. Microbes associated with black soldier fly (Diptera: Stratiomiidae) degradation of food waste. Environ Entomol. 2020;49:405–11. 31904089 10.1093/ee/nvz164

[bib54] Sheppard DC , Newton GL, Thompson SA et al. A value added manure management system using the black soldier fly. Bioresour Technol. 1994;50:275–9.

[bib55] Sontowski R , van Dam NM. Functional variation in dipteran gut bacterial communities in relation to their diet, life cycle stage and habitat. Insects. 2020;11:543. 10.3390/insects11080543 PMC7469148 32824605

[bib75_561_225122] Tegtmeier D, Hurka S, Klüber P. Cottonseed Press Cake as a Potential Diet for Industrially Farmed Black Soldier Fly Larvae Triggers Adaptations of Their Bacterial and Fungal Gut Microbiota. Front Microbiol. 2021a;12:634503. 33854488 10.3389/fmicb.2021.634503 PMC8039154

[bib76_510_225322] Tegtmeier D, Hurka S, Mihajlovic S. Culture-Independent and Culture-Dependent Characterization of the Black Soldier Fly Gut Microbiome Reveals a Large Proportion of Culturable Bacteria with Potential for Industrial Applications. Microorganisms. 2021b;9:1642. 34442721 10.3390/microorganisms9081642 PMC8398798

[bib57] Tomberlin JK , Van Huis A. Black soldier fly from pest to ‘crown jewel’ of the insects as feed industry: an historical perspective. J Insects Food Feed. 2020;6:1–4.

[bib58] Tremblay J , Singh K, Fern A et al. Primer and platform effects on 16S rRNA tag sequencing. Front Microbiol. 2015;6:771. 26300854 10.3389/fmicb.2015.00771 PMC4523815

[bib59] Ulanova RV , Tikhonova EN, Kravchenko IK. Bacteria associated with *Lucilia**sericata* larvae reared on fish wastes. Entomol Exp Appl. 2020;168:573–81.

[bib60] Wu N , Wang X, Xu X et al. Effects of heavy metals on the bioaccumulation, excretion and gut microbiome of black soldier fly larvae (*Hermetia illucens*). Ecotoxicol Environ Saf. 2020;192:110323. 32066008 10.1016/j.ecoenv.2020.110323

[bib61] Wylensek D , Hitch TC, Riedel T et al. A collection of bacterial isolates from the pig intestine reveals functional and taxonomic diversity. Nat Commun. 2020;11:1–26. 33319778 10.1038/s41467-020-19929-w PMC7738495

[bib62] Wynants E , Frooninckx L, Crauwels S et al. Assessing the microbiota of black soldier fly larvae (*Hermetia illucens*) reared on organic waste streams on four different locations at laboratory and large scale. Microb Ecol. 2019;77:913–30. 30430196 10.1007/s00248-018-1286-x

[bib64] Yang F , Tomberlin JK, Jordan HR. Starvation alters gut microbiome in black soldier fly (Diptera: Stratiomyidae) larvae. Front Microbiol. 2021;12:160. 10.3389/fmicb.2021.601253 PMC7921171 33664713

[bib63] Yang F , Tomberlin JK. Comparing selected life-history traits of black soldier fly (Diptera: Stratiomyidae) larvae produced in industrial and bench-top-sized containers. J Insect Sci. 2020;20:25. 10.1093/jisesa/ieaa113 PMC7583275 33089872

[bib65] Yarza P , Yilmaz P, Pruesse E et al. Uniting the classification of cultured and uncultured bacteria and archaea using 16S rRNA gene sequences. Nat Rev Microbiol. 2014;12:635–45. 25118885 10.1038/nrmicro3330

[bib66] Zhan S , Fang G, Cai M et al. Genomic landscape and genetic manipulation of the black soldier fly *Hermetia**illucens*, a natural waste recycler. Cell Res. 2020;30:50–60. 31767972 10.1038/s41422-019-0252-6 PMC6951338

[bib67] Zhang J , Ding X, Guan R et al. Evaluation of different 16S rRNA gene v regions for exploring bacterial diversity in a eutrophic freshwater lake. Sci Total Environ. 2018;618:1254–67. 29089134 10.1016/j.scitotenv.2017.09.228

[bib68] Zhang X , Zhang J, Jiang L et al. Black soldier fly (*Hermetia illucens*) larvae significantly change the microbial community in chicken manure. Curr Microbiol. 2020;1–13. 10.1007/s00284-020-02276-w 33141316

[bib70] Zheng L , Crippen TL, Holmes L et al. Bacteria mediate oviposition by the black soldier fly, *Hermetia**illucens* (L.),(Diptera: Stratiomyidae). Sci Rep. 2013b;3:1–8. 10.1038/srep02563 PMC3759047 23995019

[bib69] Zheng L , Crippen TL, Singh B et al. A survey of bacterial diversity from successive life stages of black soldier fly (Diptera: Stratiomyidae) by using 16S rDNA pyrosequencing. J Med Entomol. 2013a;50:647–58. 23802462 10.1603/me12199

