# Correction to: Meta-analysis of larvae of the black soldier fly (Hermetia illucens) microbiota based on 16S rRNA gene amplicon sequencing

**DOI:** 10.1093/femsec/fiad115

**Published:** 2023-11-01

This is a correction to: Freek IJdema, Jeroen De Smet, Sam Crauwels, Bart Lievens, Leen Van Campenhout, Meta-analysis of larvae of the black soldier fly (Hermetia illucens) microbiota based on 16S rRNA gene amplicon sequencing, FEMS Microbiology Ecology, Volume 98, Issue 9, September 2022, fiac094, https://doi.org/10.1093/femsec/fiac094.

In the originally published online version of this manuscript, the authors discovered an error in the 16S rRNA gene sequencing bio-informatics pipeline used: instead of using the UNOISE3 algorithm for the classification of the sequences, the UPARSE-OTU algorithm was used. As a result classic operational taxonomic units (OTUs) based on a 97% similarity cut-off were computed instead of zero-radius OTUs (zOTUs). The term “zOTU” and “zOTUs” should be read instead throughout as “OTU” and “OTUs”, both in the body of the text and headings to figures. This does not change the conclusions of the research.

In section **Introduction**, second paragraph, last part of the final sentence should read: “[…] but also to take into account microorganisms colonizing other parts in and on the insect.” instead of: “[…] but also to take into account microorganisms colonizing other parts in the insect.” In the fifth paragraph, end of third sentence and beginning of the fourth should read: “[…] with various workflows, with most studies using similarity-based clustering of the obtained sequences in ‘operational taxonomic units’ (OTUs) while others use exact[…]” instead of: “[…]with various workflows. Moreover, there is a growing tendency to replace the use of operation taxonomic units (zOTUs) based on a sequence similarity cut-off by the analysis of exact sequence variants, i.e. so-called[…]”.

In **Materials and Methods** section, under subheading **Processing of the 16S rRNA gene sequences**, second paragraph, beginning of the fourth sentence should read: “The resulting decontaminated sequences were clustered into OTUs based on a 97% identity cut-off using the UPARSE-OTU algorithm as implemented in USEARCH OTUs,[…]” instead of: “The resulting decontaminated sequences were classified into zOTUs by the UNOISE3 algorithm as implemented in USEARCH zOTUs,[…]”. The beginning of the sixth sentence should read: “Further, the identity of the most important OTUs was verified with a BLAST search of a representative sequence in GenBank[…]” instead of: “Further, the identity of the most important zOTUs was verified with a BLAST search in GenBank[…]”.

Subheading “**Factors affecting the bacterial composition of BSFL gut”** should read instead: “**Factors affecting the bacterial community composition of BSFL gut**”.

Subheading “**Difference in bacterial composition between gut and whole larval samples**” should read instead: “**Difference in bacterial community composition between gut and whole larval samples**”. Beginning of the second sentence should read: “A beta-diversity NMDS-plot[…]” instead of: “A beta-analysis NMDS-plot[…]”.

Figure 2 and its legend should read:

**Figure 2.**
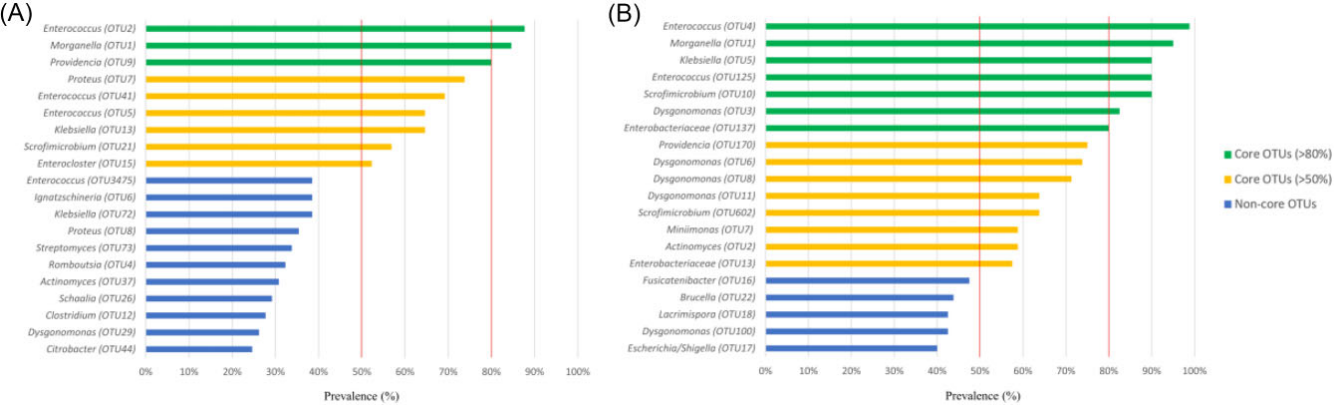
**Most prevalent OTUs in BSFL gut samples in Subsets 1 (A) and 2 (B)**. Prevalence of OTUs was based on presence and absence of OTUs across all samples analysed (n=65 for Subset 1, n=80 for Subset 2) for each separate subset. OTUs were identified using BLAST against type materials. When blasting of OTUs yielded less than 97% sequence identity with type materials, sequences were blasted against entire GenBank. OTUs were considered to be part of the BSFL core microbiota when they were prevalent in over 80% (green bars) and 50% (yellow bars) of all samples within each group. Blue bars represent prevalent species under these threshold values.

instead of:

**Figure 2.**
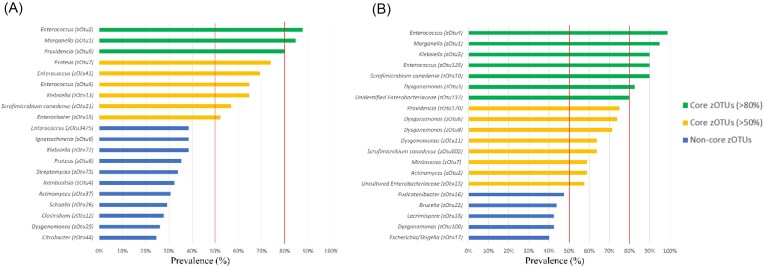
**Most prevalent zOTUs in BSFL gut samples in Subsets 1 (A) and 2 (B)**. Prevalence of zOTUs was based on presence and absence of zOTUs across all samples analysed (n=65 for Subset 1, n=80 for Subset 2) for each separate subset. zOTUs were identified using BLAST against type materials. When blasting of zOTUs yielded less than 97% sequence identity with type materials, sequences were blasted against entire GenBank. zOTUs were considered to be part of the BSFL core microbiota when they were prevalent in over 80% (green bars) and 50% (yellow bars) of all samples within each group. Blue bars represent prevalent species under these threshold values.

Subheading “**Experimental parameters affecting the gut bacterial composition**” should read instead: “**Experimental parameters affecting the gut bacterial community composition”**. In the first paragraph, second part of the fifth sentence should read: “[…]which in turn may have been reflected in the microbial community composition of the larvae” instead of: “[…]which in turn may have been reflected in the microbial composition of the larvae”.

In section **Challenges in performing a meta-analysis of the BSFL microbiota**, end of the second paragraph should read additionally: “Furthermore, future studies would benefit from a consensus on the use of OTUs or ASVs. While 97% OTUs minimize effects of sequencing errors, ASVs increase taxonomic resolution but require critical denoising methods to obtain robust sequence data (Callahan et al. 2017; Edgar, 2016).”

These errors have been outlined only in this correction notice to preserve the version of record.

