# Supplementary material for: Meta-analysis of larvae of the black soldier fly (Hermetia illucens) microbiota based on 16S rRNA gene amplicon sequencing
Source: FEMS Microbiol Ecol. 2022 Aug 17;98(9):fiac094. doi: 10.1093/femsec/fiac094 (PMC9453823; doi:10.1093/femsec/fiac094)
Supplement: fiac094_Supplemental_Files [file fiac094_supplemental_files.zip › Legends_Supplementary_figures.docx]

**Legends supplementary figures**

**Supplementary Figure S1. Rarefaction curves of Subset 1.** Colors indicate from which study the sample originates from.

**Supplementary Figure S2. Rarefaction curves of Subset 2.** Colors indicate from which study the sample originates from.

**Supplementary Figure S3. Rarefaction curves of Subset 3.** Colors indicate from which study the sample originates from.

**Supplementary Figure S4. Total number of reads from four zOTUs from Subset 2, all identified as members of the genus Dysgonomonas.** The x-axis shows the feed types used in the three separate studies, while the y-axis shows the total number of reads for each zOTU. In the Cifuentes et al. (2020) dataset, only zOTU8 was found with a total of 206 reads.

**Supplementary Figure S5. Overview of all samples and relative abundance of the core zOTUs of Subset 1.** Reads from zOTUs other than the core bacteria are clustered together as “Other”. Samples from the study of Tegtmeier et al. (2021a) are highlighted, starting with the identifiers SRR1300. Red encircled samples were reared on a cottonseed press cake substrate, green encircled samples were reared on a control chicken diet.

**Supplementary Figure S6. Overview of all samples and relative abundance of the core zOTUs of Subset 2.** Reads from zOTUs other than the core bacteria are clustered together as “Other”.
