# Supplementary figures and images for: Meta-analysis of larvae of the black soldier fly (Hermetia illucens) microbiota based on 16S rRNA gene amplicon sequencing
Source: FEMS Microbiol Ecol. 2022 Aug 17;98(9):fiac094. doi: 10.1093/femsec/fiac094 (PMC9453823; doi:10.1093/femsec/fiac094)

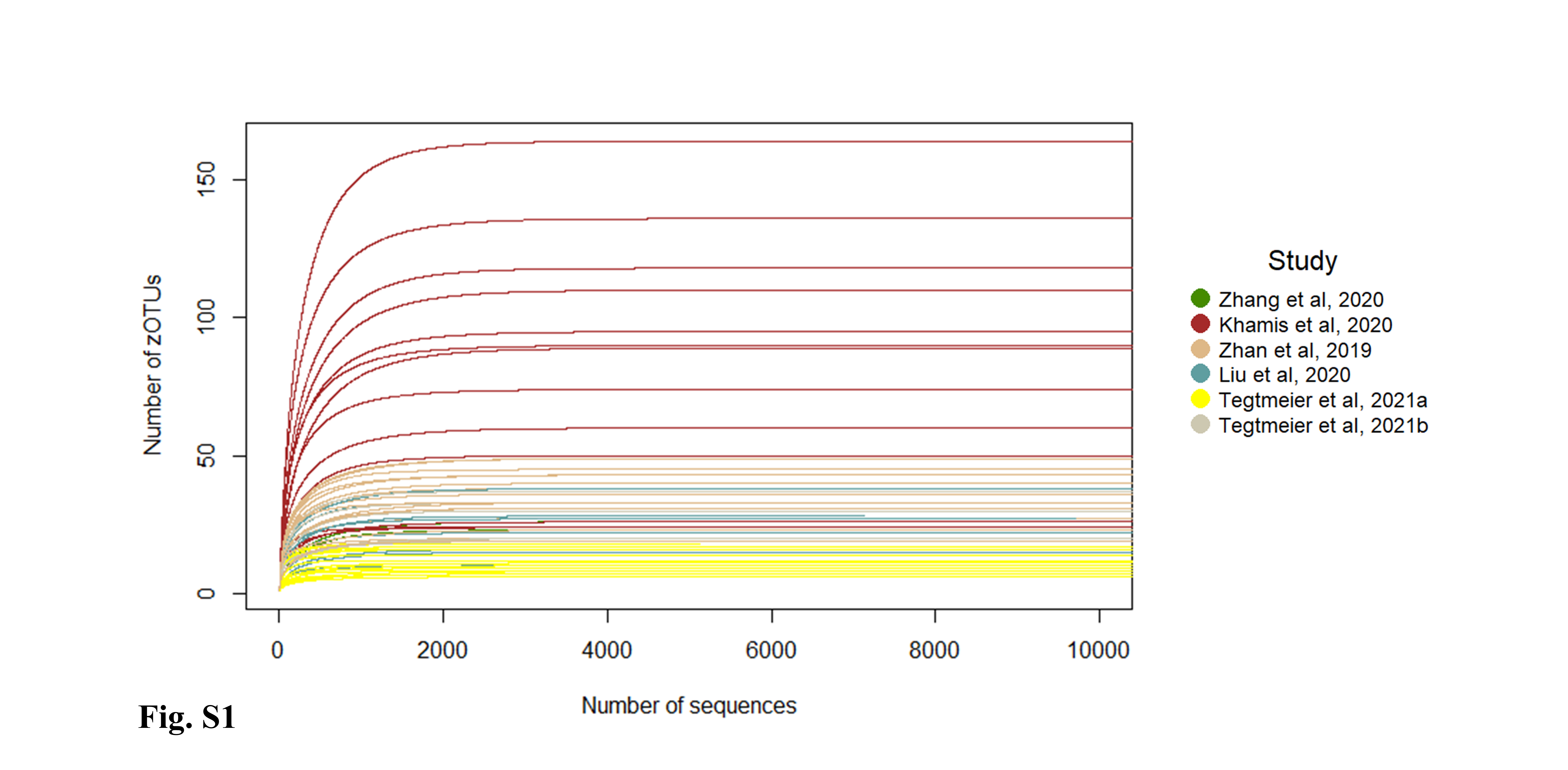

Supplement: fiac094_Supplemental_Files [file fiac094_supplemental_files.zip › Supplementary_Figure_S1.tif]

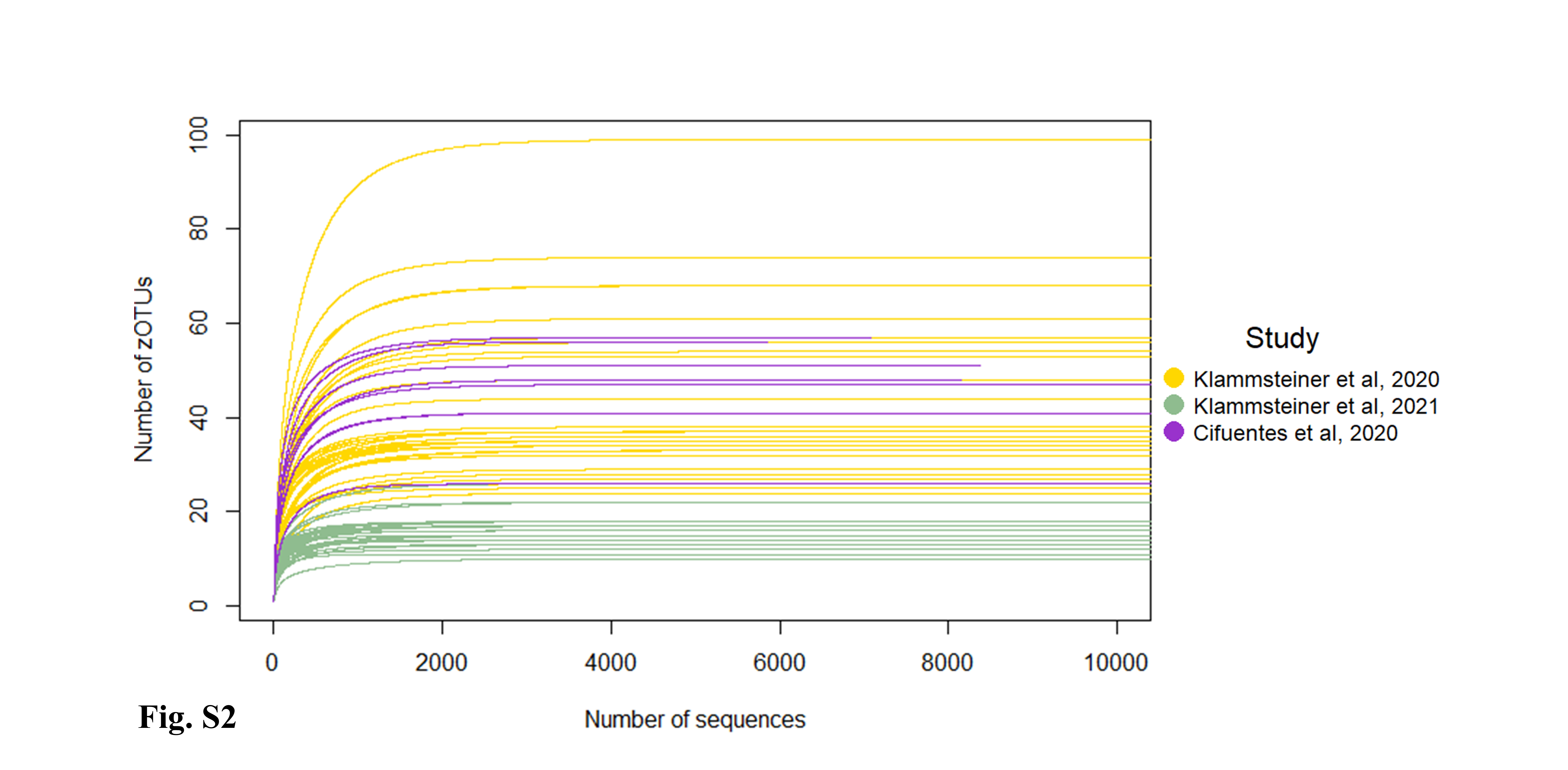

Supplement: fiac094_Supplemental_Files [file fiac094_supplemental_files.zip › Supplementary_Figure_S2.tif]

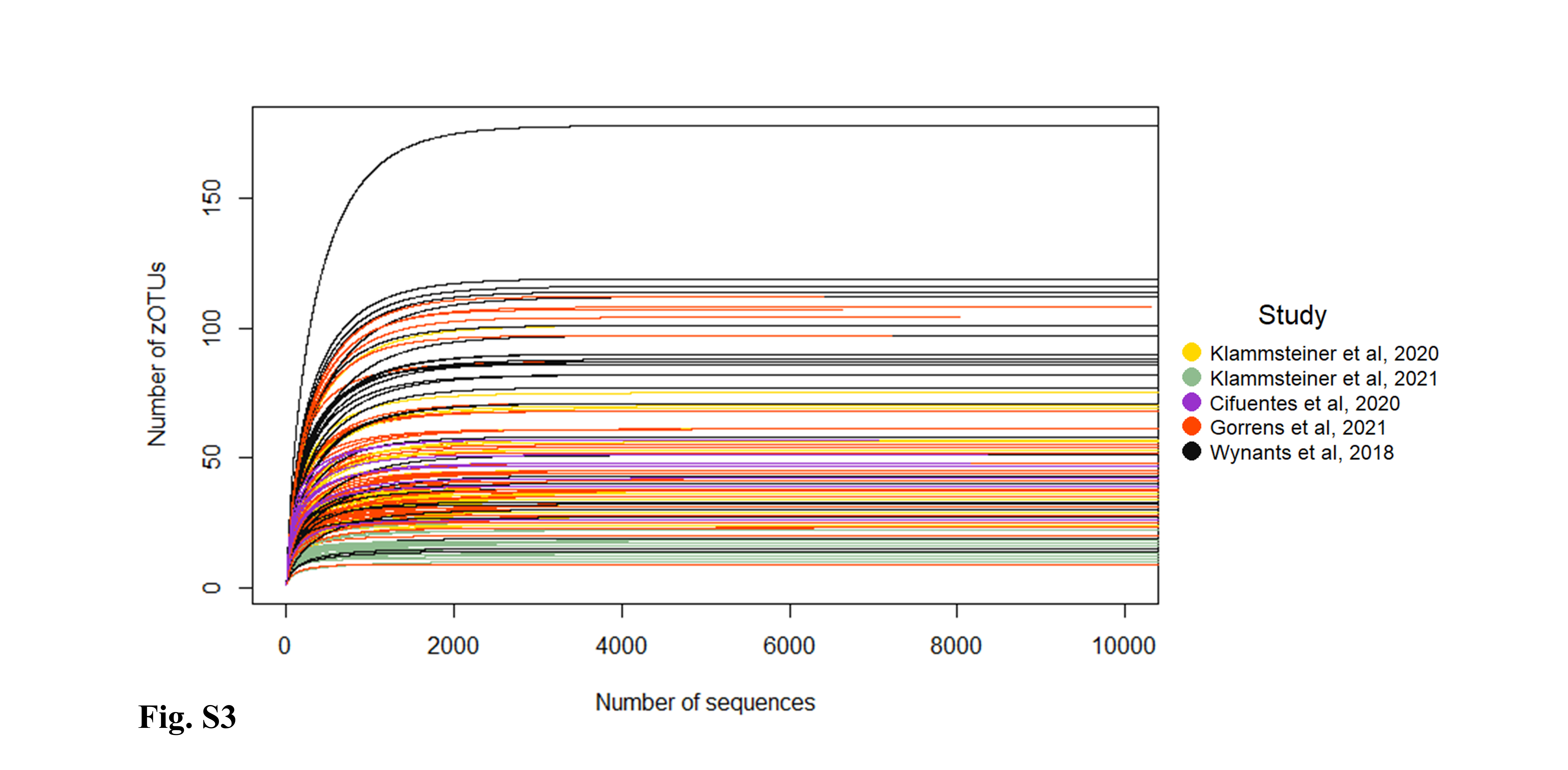

Supplement: fiac094_Supplemental_Files [file fiac094_supplemental_files.zip › Supplementary_Figure_S3.tif]

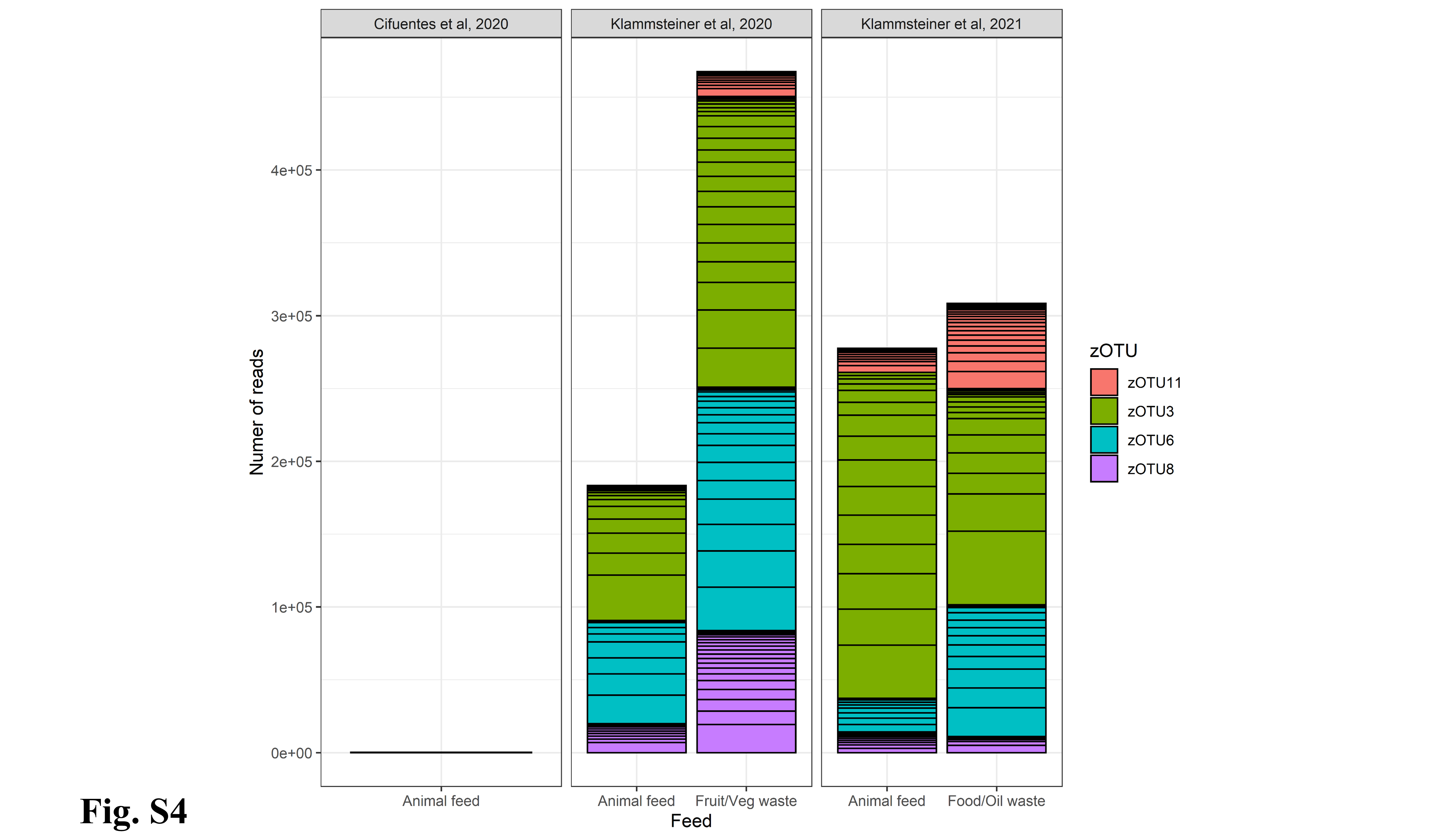

Supplement: fiac094_Supplemental_Files [file fiac094_supplemental_files.zip › Supplementary_Figure_S4.tif]

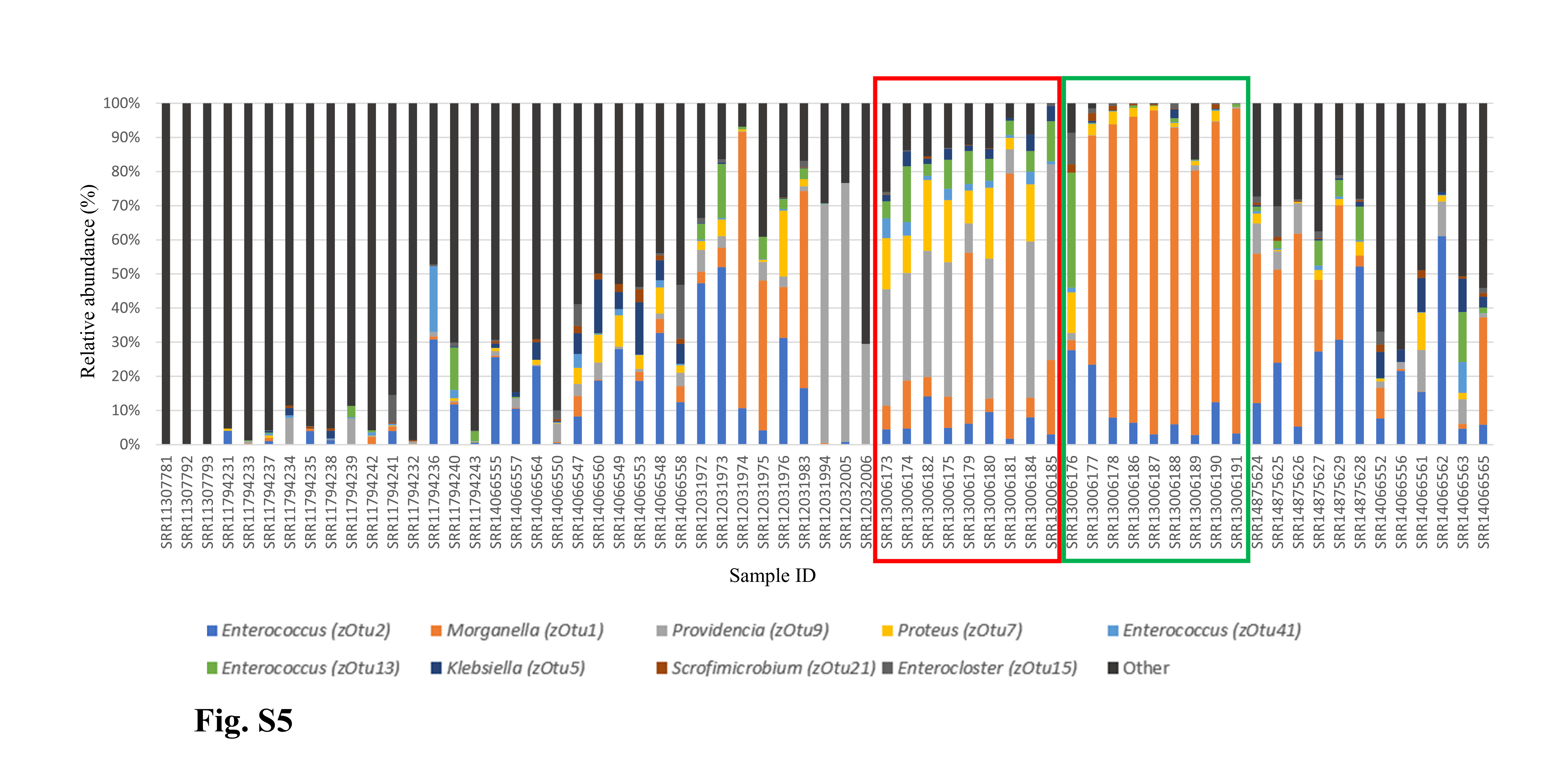

Supplement: fiac094_Supplemental_Files [file fiac094_supplemental_files.zip › Supplementary_Figure_S5.tif]

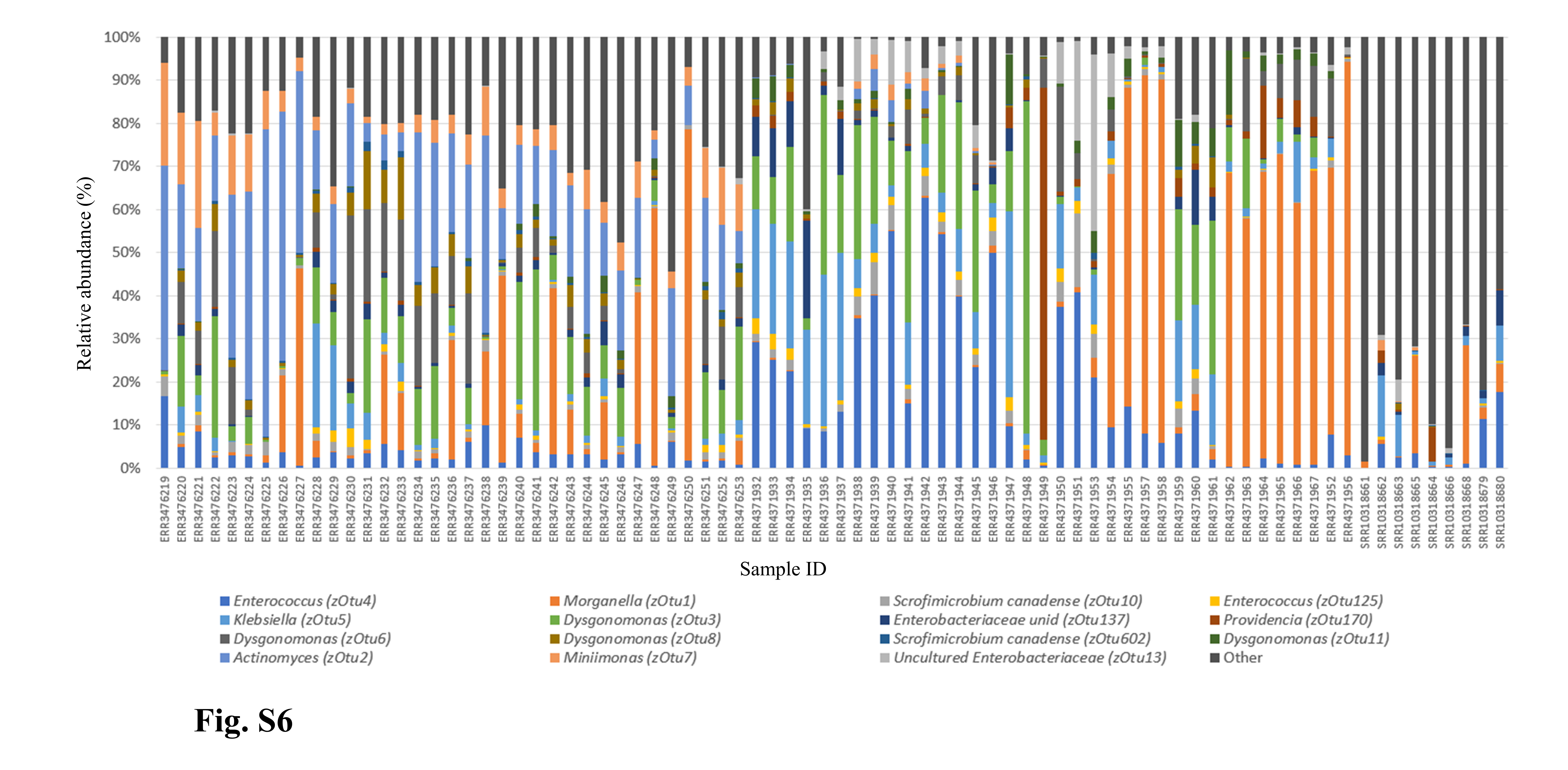

Supplement: fiac094_Supplemental_Files [file fiac094_supplemental_files.zip › Supplementary_Figure_S6.tif]
